# Cell membrane-camouflaged inorganic nanoparticles for cancer therapy

**DOI:** 10.1186/s12951-022-01475-w

**Published:** 2022-06-18

**Authors:** Wanli Song, Pengfei Jia, Ting Zhang, Keke Dou, Lubin Liu, Yaping Ren, Fujun Liu, Junmiao Xue, Mohamed Sayed Hasanin, Hongzhao Qi, Qihui Zhou

**Affiliations:** 1 Institute for Translational Medicine, Department of Stomatology, The Affiliated Hospital of Qingdao University, Qingdao University, Qingdao, 266003 China; 2 School of Stomatology, Qingdao University, Qingdao, 266003 China; 3 Cellulose and Paper Department, National Research Centre, Dokki, 12622 Cairo Egypt

**Keywords:** Cell membrane, Inorganic nanoparticles, Cancer therapy, Active targeting, Multifunction nanoplatform

## Abstract

Inorganic nanoparticles (INPs) have been paid great attention in the field of oncology in recent past years since they have enormous potential in drug delivery, gene delivery, photodynamic therapy (PDT), photothermal therapy (PTT), bio-imaging, driven motion, etc. To overcome the innate limitations of the conventional INPs, such as fast elimination by the immune system, low accumulation in tumor sites, and severe toxicity to the organism, great efforts have recently been made to modify naked INPs, facilitating their clinical application. Taking inspiration from nature, considerable researchers have exploited cell membrane-camouflaged INPs (CMCINPs) by coating various cell membranes onto INPs. CMCINPs naturally inherit the surface adhesive molecules, receptors, and functional proteins from the original cell membrane, making them versatile as the natural cells. In order to give a timely and representative review on this rapidly developing research subject, we highlighted recent advances in CMCINPs with superior unique merits of various INPs and natural cell membranes for cancer therapy applications. The opportunity and obstacles of CMCINPs for clinical translation were also discussed. The review is expected to assist researchers in better eliciting the effect of CMCINPs for the management of tumors and may catalyze breakthroughs in this area.

**Graphical Abstract:**

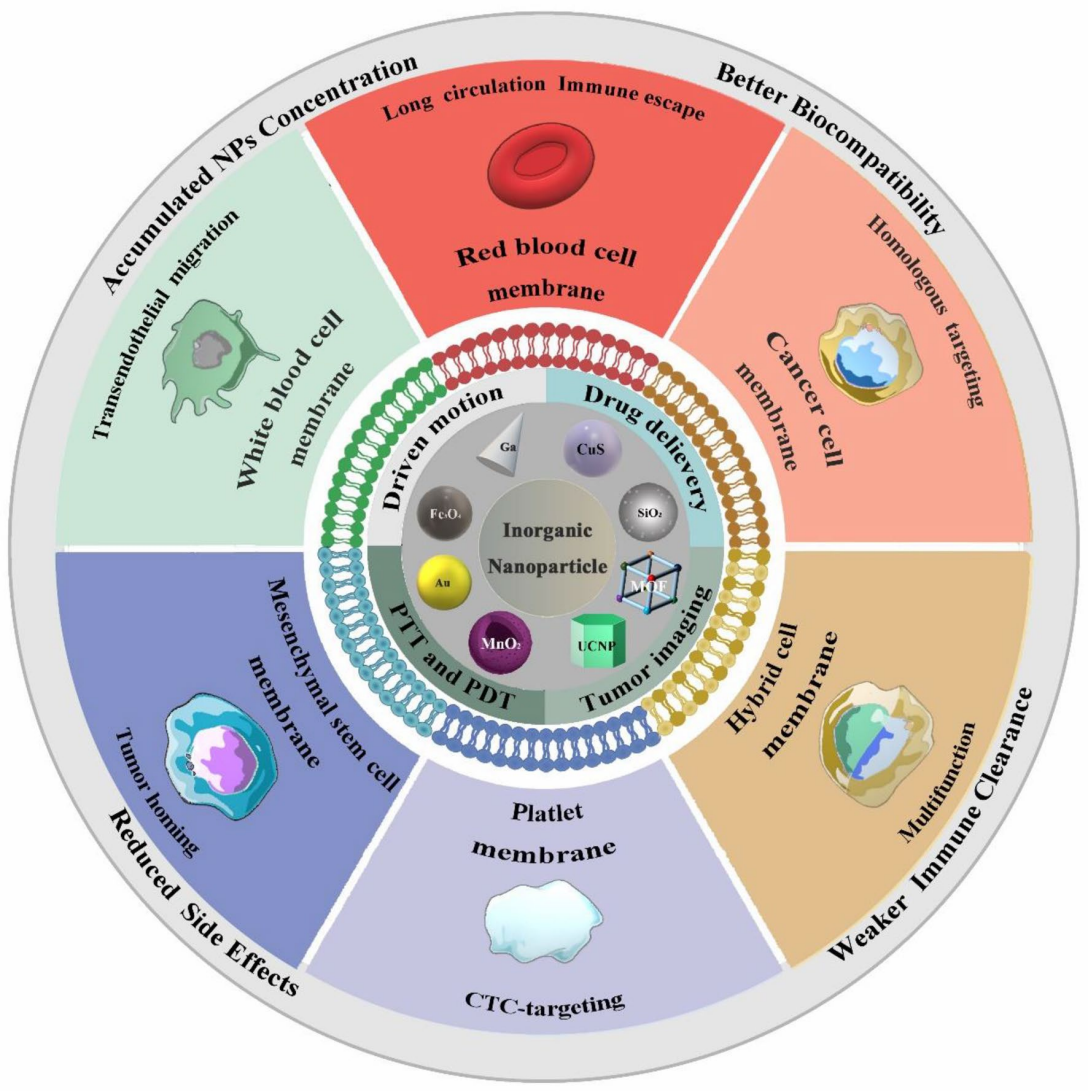

## Introduction

Cancer is the second leading cause of death and has been doing great harm to human health [1–4]. There are an estimated 19.3 million [95% uncertainty interval (UI): 19.0–19.6 million] new cases of cancer (18.1 million excluding non-melanoma skin cancer) and almost 10.0 million (95% UI: 9.7–10.2 million) deaths from cancer (9.9 million excluding non-melanoma skin cancer) worldwide in 2020 [5]. Conventional methods to treat cancer include surgery, chemotherapy, and radiotherapy. However, if tumors are non-resectable or metastasized, chemotherapy is the only therapeutic resolution to control the size and spread of cancer. As one of the most common clinical strategies, chemotherapy still has unsatisfactory performance due to severe adverse effects and the low targeting ability of anti-cancer drugs [6, 7]. To deal with the difficulties above, targeting drug delivery systems (TDDS) based on nanoparticles (NPs), including organic NPs (ONPs) and inorganic NPs (INPs), have been widely developed [8–13]. Taking advantage of their unique (bio)physicochemical characteristics, such as small size (100–200 nm) and optimized surface potential (negative or neutral), NPs can enhance the accumulation of anti-cancer drugs in cancers and reduce their side effects [14]. Compared with ONPs, INPs possess unique optical, electric, and magnetic characteristics, making them have a promising application prospect in tumor diagnosis and treatment [15–21]. However, the clinical application of INPs remains a considerable challenge because of their limited biocompatibility and targeting ability. For instance, the naked INPs are readily captured by the reticuloendothelial system (RES) due to their exogenous source [22]. To date, a significant amount of effort has been dedicated to exploring methods to endow INPs with better biocompatibility and targeting ability. Surface modification of INPs is one of the most commonly used methods. One typical example is that polyethylene glycol (PEG) modification (PEGylation) could reduce the elimination of INPs by the RES to prolong their blood circulation time [23]. Furthermore, targeting moieties can be further immobilized on PEGylated INPs to improve their targeting efficiency. However, surface modification can not completely avoid the recognition of immune systems because the PEGylation can not reach the whole coverage of INPs to form an integrated protective shell. As an example, an immune response can be induced by the repetitive administration of PEGylated INPs, dramatically reducing their biocompatibility [24, 25]. Therefore, it is urgently needed to exploit more robust methods to modify INPs for their clinical application.

**Fig. 1:**
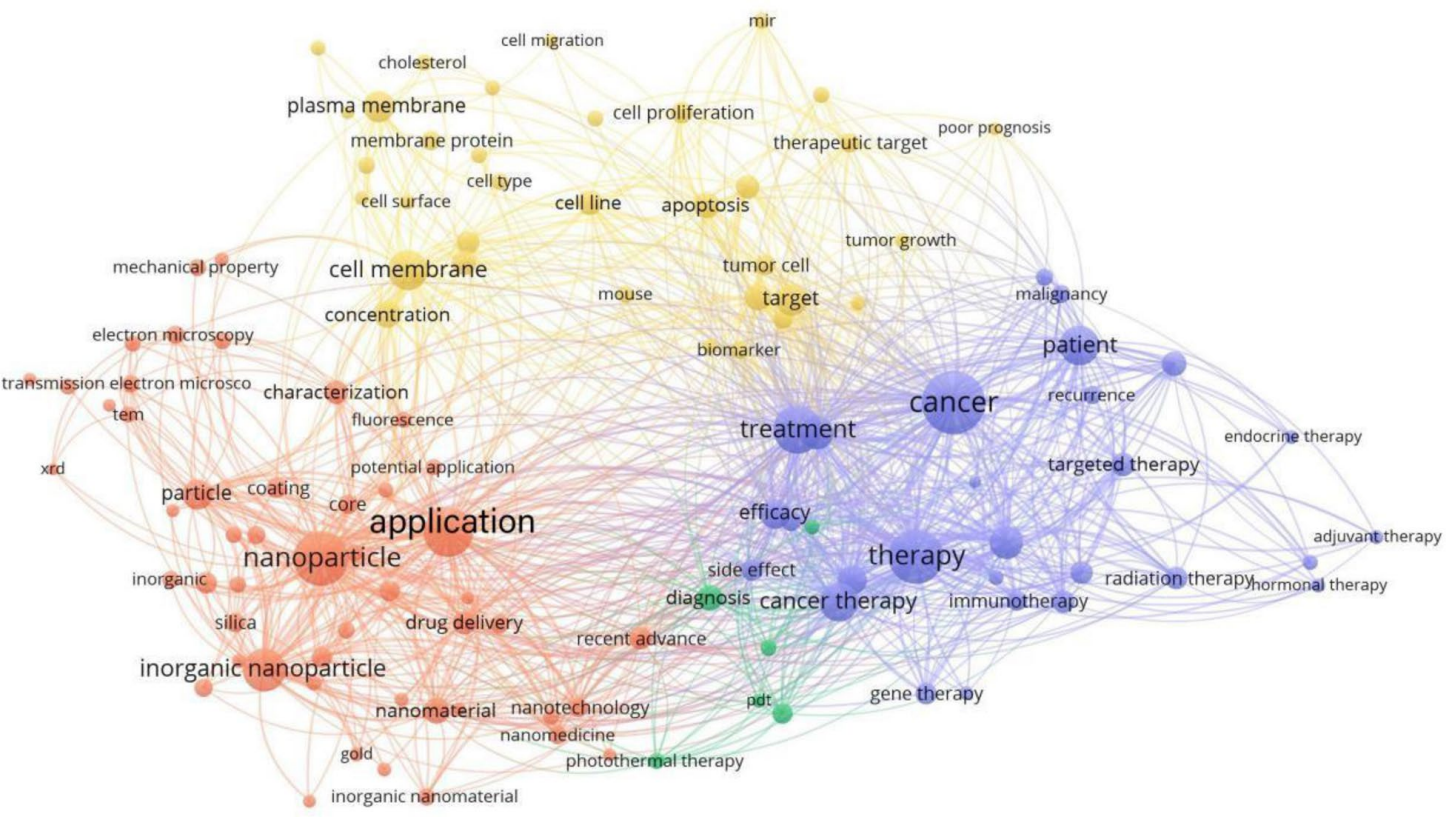
The analysis of keyword co-occurrences on inorganic nanoparticles, cell membrane, cancer therapy, cancer target, and multifunction nanoplatform

An entire area of research dedicated to biomimetic nanotechnologies has spawned in recent decades, and it provides a new approach for the modification of INPs. Researchers have recently got the idea from nature to fabricate biomimetic cell membrane-camouflaged nanoparticles (CMCNPs), and the cell membrane can cover the whole surface of NPs to avoid potential immune responses like PEGylated NPs. Initially, the CMCNP was designed as a core–shell nanocomposite by co-extrusion on a mini-extruder with red blood cell (RBC) membrane and poly lactic-co-glycolic acid (PLGA), a kind of ONPs. Subsequently, diverse cell membrane-camouflaged inorganic NPs (CMCINPs) were explored, combining the strengths of a cell membrane with the INPs [26, 27]. The INPs can be disguised as endogenous substances to escape elimination from the immune system and prolong their blood circulation time, which is extremely necessary for tumor targeting [28]. Moreover, the functional ingredients on the surface of the natural cell membrane, such as simple sugars, peptides, proteins and so on, can be maintained in CMCINPs. All these properties can endow the INPs with some biological functions as a biomimetic nanoplatform. It has been reported that numerous cells are involved in the development and therapy of cancer, such as white blood cells, platelets, cancer cells, and mesenchymal stem cells [29]. Extravasation, cytokine chemotaxis, and cancer cell adhesion, which are the cell membrane-based functions of cancer-related cells, inspired researchers to explore the CMCINPs as a nanoplatform for drug delivery system, bio-imaging, diagnostic agents, etc*.* [30, 31]. In recent years, CMCINPs have been developed to be a flourished anti-cancer nanoplatform field. The VOS viewer bibliometric visualization software is used to analyze co-occurrences on INPs, cell membrane, cancer therapy, cancer target, and multifunction nanoplatform (Fig. 1). The report of scientific researches shows that the combination of INPs and cell membranes has a broad application for cancer therapy, giving researchers a hint to explore new strategies based on CMCINPs to fight against cancer, such as immunotherapy and radiation therapy.

**Fig. 2:**
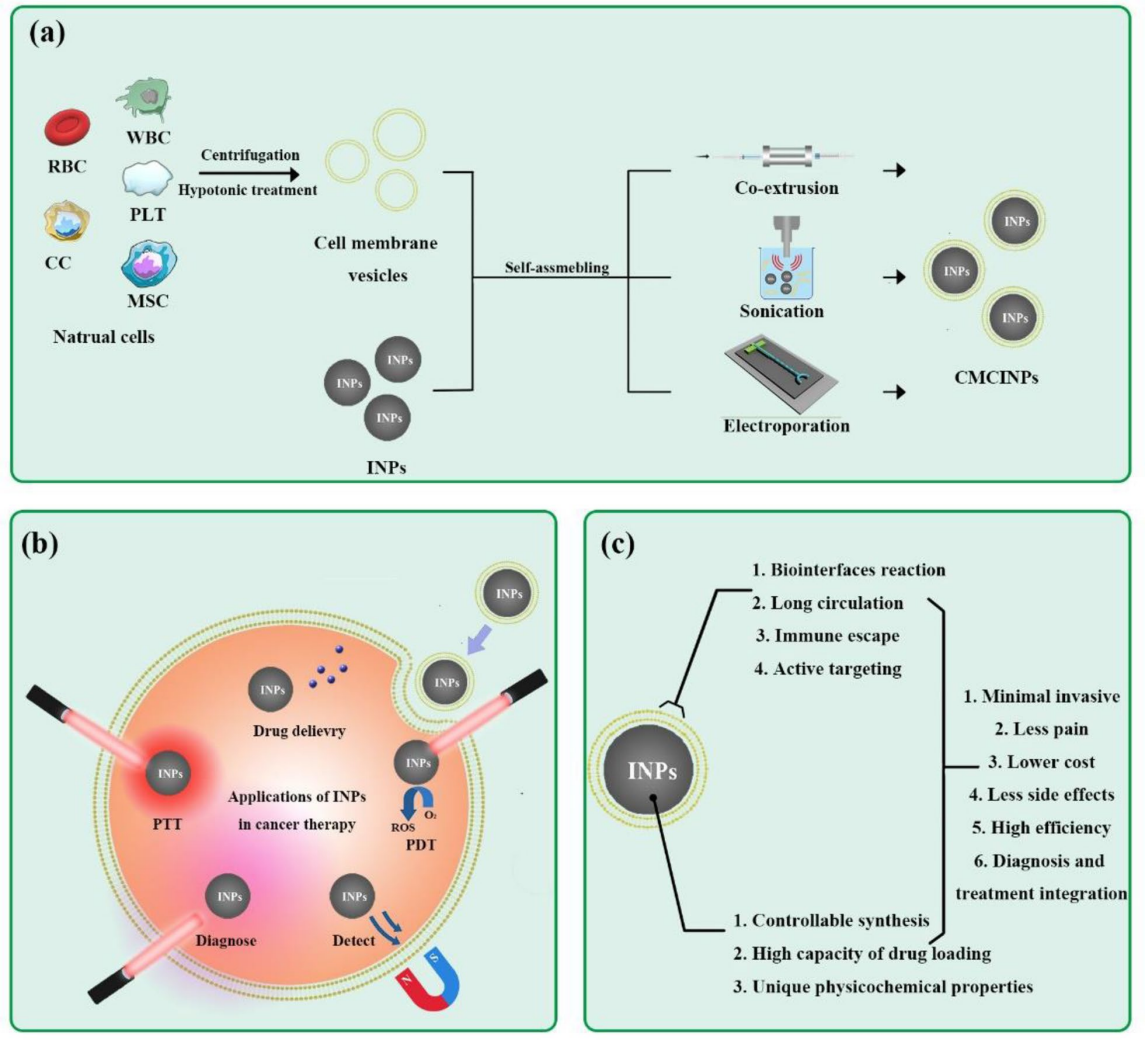
**a** Scheme of the preparation of CMCINPs. **b** Applications of CMCINPs in cancer therapy, including drug delivery, PDT, PTT, diagnosis, and magnetic driven targeting. **c** Advantages of CMCINPs in cancer therapy

Generally, the formation of CMCINPs follows the method as shown in Fig. 2a. The cell membrane is separated and broken into fragments to make cell membrane vesicles in a uniform size. Then the INPs are camouflaged by various cell membrane vesicles to form CMCINPs. The technologies for assembling CMCINPs are detailedly introduced in our review. Cell membranes from various cells can endow CMCINPs with different functions, resulting in diverse biological behaviors both in vitro and in vivo. Besides the versatile capacity of the camouflaged membrane, the inner INPs of CMCINPs are also fantastic candidates for various cancer therapy-related applications, such as drug delivery, bio-imaging, magnetic field driving, photothermal therapy (PTT), photodynamic therapy (PDT), etc. (Fig. 2b) [32]. The advantages of CMCINPs are also listed in Fig. 2c. Moreover, we discuss the prospects and challenges of biomimetic cancer therapeutic nanoplatforms for clinic applications in the future.

## Technologies for assembling CMCINPs

Multifunctional inorganic nanoplatforms camouflaged with natural cell membranes usually include three main steps to achieve self-assembling: (1) separation of cell membrane-derived vesicles, (2) synthesis of the inorganic nanoplatform cores, and (3) final assembling cell membrane vesicles and INPs together to form a core–shell nanostructure [33]. The third phase is undoubtedly the most vital procedure to determine the successful preparation of CMCINPs. The process of camouflaging should be efficient, but it should also prevent drug leakage and protein denaturation. Recent research has developed some new preparation methods for the last phase: co-extrusion, sonication microfluidic electroporation, spontaneous formation by electrostatic attractions, in situ polymerization, and graphene nanoplatform-mediated cell membrane coating [32]. Herein, we firstly give a detailed introduction to methods for cell membrane-derived vesicles separation, and then focus on the three most frequently used technologies for assembling CMCINPs as follows.

### Methods for cell membrane-derived vesicles separation

The separation of cell membrane-derived vesicles includes a series of procedures as follows: (**i)** The sufficient quantities of source cells ( 200–300 million cells) are required for cell membrane extraction to assemble CMCINPs [34]. (ii) Hypotonic treatment or freeze–thaw cycle is used for cell lysis. (iii) Differential centrifugation is applied for discarding the nucleus and other intracellular contents. (iv) Washed with phosphate-buffered saline (PBS) several times containing protease inhibitor for purifying the cell membrane fragments. (v) Disperse the cell membrane fragments in PBS or other mild dispersion. (vi) Homogenize the cell membrane fragments dispersion by extrusion using a mini-extruder with porous polycarbonate membrane for forming uniform-sized cell membrane-derived vesicles, and finally, (vii) save in 4 ℃ for further use (better to use it right away in case denaturation of functional protein ligands) [36].

During the cell membrane-vesicles separation process, there could be a loss of structural components like cholesterol and functional components like cell membrane protein ligands/receptors. The rigidity of the cell membrane mainly relies on cholesterol, so the loss of cholesterol may decrease the mechanical stability of the cell membrane [35], making it unstable for assembling CMCINPs. In order to maintain the cell membrane stability and decrease the loss of functional proteins, it is considered to add cholesterol and divalent ions (like MgCl_2_, CaCl_2_, etc*.*) to the hypotonic buffers [34]. Meanwhile, rigorous operation conditions and sequence are also crucial to prevent the degradation of the functional surface cell membrane protein ligands/receptors, such as ice-bath conditions, mild lysis hypotonic buffers, and appropriate rupture techniques. It is worth mentioning that the cell membrane-derived vesicles should be used right away or saved in 4 ℃ for further use.

#### Co-extrusion

Extrusion is the process of extruding material through a porous membrane with a specific cross-sectional area to produce particles of uniform size [37]. To prepare CMCINPs, the mixed solution of cell membrane vesicles and INPs was passed through a porous polycarbonate membrane over and over again in a small extruder. Because of the fluidity of the cell membrane, the mechanical force applied during extrusion promotes INPs to cross the phospholipid bilayers and helps the cell membrane to wrap around INPs, leading to a vesicle-nanoparticle fusion. Many studies have reported the technology for assembling CMCINPs by using polycarbonate membranes with different pore sizes [38]. Because of the uniform diameters of membrane pores, the uniform size of the resultant CMCINPs could be fabricated. Moreover, the prepared CMCINPs exhibit a uniform distribution by extrusion, which assures the quality of CMCINPs for further use. In fact, since the technology is based on mechanical force instead of the chemical synthesis process, it could remain the surface protein of cell membranes to a large extent, which ensures the bio-activity of cell membranet [32]. However, the chief limitation of this method is the loss of samples as a result of INPs would inevitably accumulate on the porous membrane during the extruding process, which results in the difficulty of large-scale production. Futhermore, it can be a cumbersome and time-consuming process, limiting its clinical application.

#### Sonication

Compared to co-extrusion, sonication is an effective alternative. Cavitation bubbles which are generated by ultrasonic waves can destroy the structure of the membrane and accomplish the recombination of cell membrane fragments around INPs. The noncovalent interactions between INPs and cell membranes are also helpful to the fusion of each other. Compared with the extrusion method, by optimizing the parameters such as input power, frequency, and time, ultrasound can avoid the damage of cell membrane or protein denaturation to a certain extent caused by heat energy in the fusion process. It only needs one step to fabricate, which is facile and time-saving. In addition, the asymmetric surface charge of the cell membrane and the stable nature of cell membrane vesicles and INPs core can promote the process of cell membrane fusion [39]. However, the resulting particles may have different sizes and be featured by the uniformity of the cell membrane shell.

#### Microfluidic electroporation

Electroporation is a high-throughput technology that integrates nanoparticles into cells [38]. The typical microfluidic chip for electroporation is composed of a Y-shaped merging channel, an S-shaped mixing channel, an electroporation area, two inlets, and one outlet**.** Cell membrane vesicles and INPs were injected into the microfluidic chip from two inlets severally. Then they were fully mixed in the S-shaped channel. During the mixture flows passing through the electroporation zone, the rapid high-voltage electric field pulse produces multiple instantaneous pores on the cell membrane for INPs to enter. The electroporation device is integrated with a microfluidic chip with an S-shaped channel, which promotes to mix efficiently INPs fed through the Y-shaped microchannel and cell membrane vesicles. This technology revealed obvious superiority in reducing protein loss on the surface of the cell membrane and maintaining cell membrane integrity [40]. By fine-tuning some parameters, this method can prepare high-quality INPs with complete cell membrane coating and excellent stability. This technique possesses unparalleled merits such as high throughput, quantitative determination, and fine parallelism. However, the cost of this technology is slightly more expensive than the first two techniques.

## Red blood cell membrane-camouflaged inorganic nanoparticles (RBCM-INPs)

Red blood cell (RBC) is the most abundant cell component with the longest circulation time in the blood [41]. The annual clinical blood transfusion volume is up to 50 million units approximately, which makes RBC widely available [42]. Moreover, the RBC membrane is readily extracted and purified since mature RBC lacks nuclei and organelles, facilitating it as a coating material for drug delivery systems [43]. To the best of our knowledge, the RBC membrane is the first cell membrane type used to coat NPs, and the resulting delivery systems show extensive properties, such as immune evasion, excellent stability, sustained and controllable drug release, prevention of forming protein-corona, and resistance to complement reaction [41, 44]. To explain the immune evasion, the RBC membrane presents a surface protein-CD47, which is also named as the ‘do not eat me’ marker. CD47 can specifically bind to one type of macrophage membrane surface protein called signal-regulatory protein alpha glycoprotein to prevent its uptake [44].

For the first time, Zhang et al*.* developed the technology of camouflaging NPs with RBC membrane and verified that the doxorubicin (DOX), a kind of chemotherapeutic drug, was successfully loaded into the PLGA core followed by camouflaging with RBC membrane [41]. Compared with the free DOX and the PLGA core, the RBC membrane-camouflaged group exhibited more powerful tumor growth inhibition and excellent reliable immune compatibility, which brought a new idea to chemotherapy. In recent years, numerous INPs, like Fe_3_O_4_ NPs, upconversion NPs (UCNPs), mesoporous silica NPs (MSNs), and plasmonic gold NPs (AuNPs), have also been modified by the coating of RBC membrane. Herein, we give a comprehensive introduction to tumor-targeting therapies based on RBCM-INPs.

**Fig. 3:**
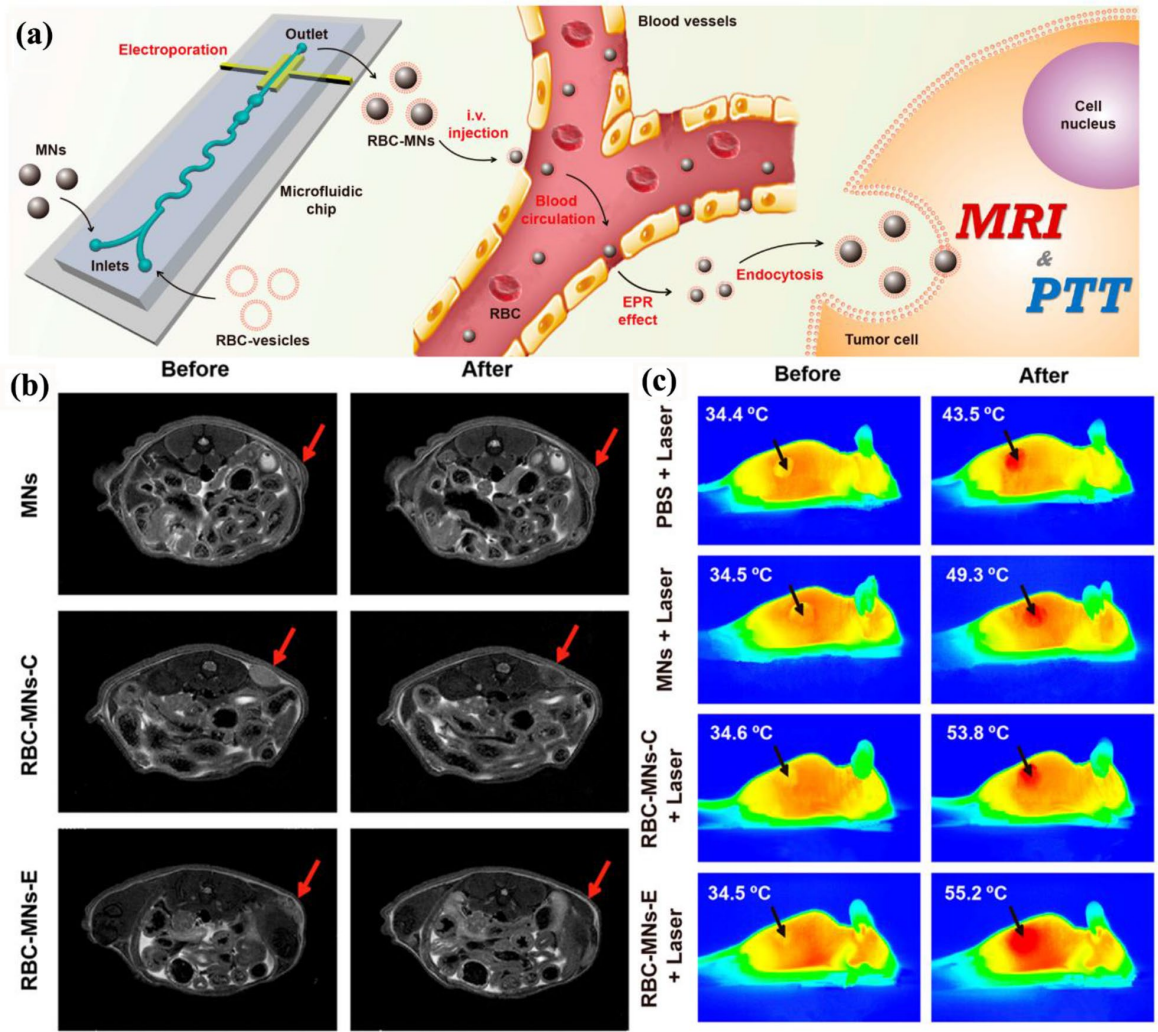
**a** Microfluidic electroporation-facilitated synthesis of RBC-MNs for enhanced imaging-guided cancer therapy. **b** Representative in vivo T_2_-weighted MRI images of tumor-bearing mice before and after the injection of PBS or PBS containing NPs. Red arrows indicate the tumor sites. **c** Representative in vivo IR thermal images of tumor-bearing mice before and after the treatment. Black arrows indicate the tumor sites Reprinted with permission from Refs [48]

Due to the unique magnetism, easily controlled size, good biodegradability, and excellent biocompatibility, Fe_3_O_4_ NPs have been proved to be suitable for MRI, anti-tumor drug delivery, hyperthermia, and tissue repair. In the past decades, plenty of studies have focused on the diagnosis and therapy application of Fe_3_O_4_ NPs [45, 46]. Rao et al*.* designed the RBC membrane-camouflaged Fe_3_O_4_ NPs (Fe_3_O_4_@RBC) by co-extrusion and demonstrated that Fe_3_O_4_@RBC was superior to PEG-modified Fe_3_O_4_ NPs (Fe_3_O_4_@PEG) to achieve long circulation by reducing the uptake by macrophage and organs rich in RES, such as spleen and liver [47]. This excellent work revealed that RBC membrane-coating was superior to the gold standard of PEG-modified NPs for ‘camouflaging’. Moreover, Rao et al*.* firstly utilized microfluidic electroporation technology to assemble RBC membrane-camouflaged clustered Fe_3_O_4_ magnetic NPs (RBC-MNs-E) to achieve better membrane coating than the co-extrusion method (RBC-MNs-C), which were further applied for enhanced tumor PTT and MRI (Fig. 3a) [48]. In this work, the tumor-bearing mice in different groups were treated with intravenous injection of clustered Fe_3_O_4_ magnetic NPs (MNs), RBC-MNs-C, or RBC-MNs-E to observe the biodistribution and performance of NPs in vivo. The mice were used for MRI tests before and after 24 h of the injection. As shown in Fig. 3b, a distinct and clear tumor darkening was observed in the tumor site after the injection of RBC-MNs-E compared with uncoated MNs and RBC-MNs-C, which means RBC-MNs-E accumulated the most in the tumor site. To evaluate the photothermal conversion efficiency, another group of mice was injected with MNs, RBC-MNs-C, and RBC-MNs-E, and PBS was used as a control. The mice received RBC-MNs-E + laser got a temperature increase from 34.5 to 55.2 ℃ in the tumor site within 5 min, which outperformed the RBC-MNs-C + laser group (Fig. 3c). The ability to eliminate tumors was also investigated in the following experiments, demonstrating the advantage of microfluidic electroporation technology. With an external magnetic field upon the tumor site, the NPs accumulation would be much higher to achieve better anti-tumor efficiency. This work verified that microfluidic electroporation is superior to the co-extrusion method to prepare CMCINPs for cancer therapy, which may be attributed to its superiority in reducing cell membrane protein loss and maintaining the integrity of the membrane to form high-quality INPs with complete cell membrane coating and excellent stability.

PDT using upconversion nanoparticles (UCNPs) is a convenient and robust approach for tumor imaging and treatment, which can cause oxidative damage to tumor cells by generating reactive oxygen species (ROS). Compared with traditional down-conversion fluorescent nano-probes, UCNPs have excellent properties, such as high light conversion reactivity, making UCNP-based PDT more efficient than other cancer treatment methods such as surgery and chemotherapy [49]. Consequently, UCNPs modified with ligands would be a great choice for active cancer-targeting for imaging and treatment. However, UCNPs in the live internal environment are facile to attract plasma proteins and form a protein-nanoparticle compound, also called “protein corona”. The compound would cover the modified targeting ligands on UCNPs’ surfaces and remarkably undermines the UCNPs’ targeting ability and biocompatibility [50]. Ding et al*.* first demonstrated that after the UCNPs were camouflaged by the RBC membrane, the UNCPs would hardly adsorb any proteins on the surface when exposed to human plasma [51]. Furthermore, they demonstrated that cell membrane coating rescued the cancer-targeting ability of folic acid (FA)-functionalized nanoparticles (FA-RBCM-UCNPs). The FA-RBCM-UCNPs were confirmed reliable for enhancing tumor imaging in the following experiment sections. Moreover, the systematic toxicity of the camouflaging biomimetic FA-RBCM-UCNPs was also investigated by blood parameters and histology analysis to assure excellent biocompatibility. This study inspired more researchers to modify and engineer cell membranes to endow CMCINPs with more functions. However, it is worth mentioning that though modification overcomes the dilemma of single functions of natural cell membranes, the modification process needs optimization since chemical reaction is hard to control to some degree.

**Fig. 4:**
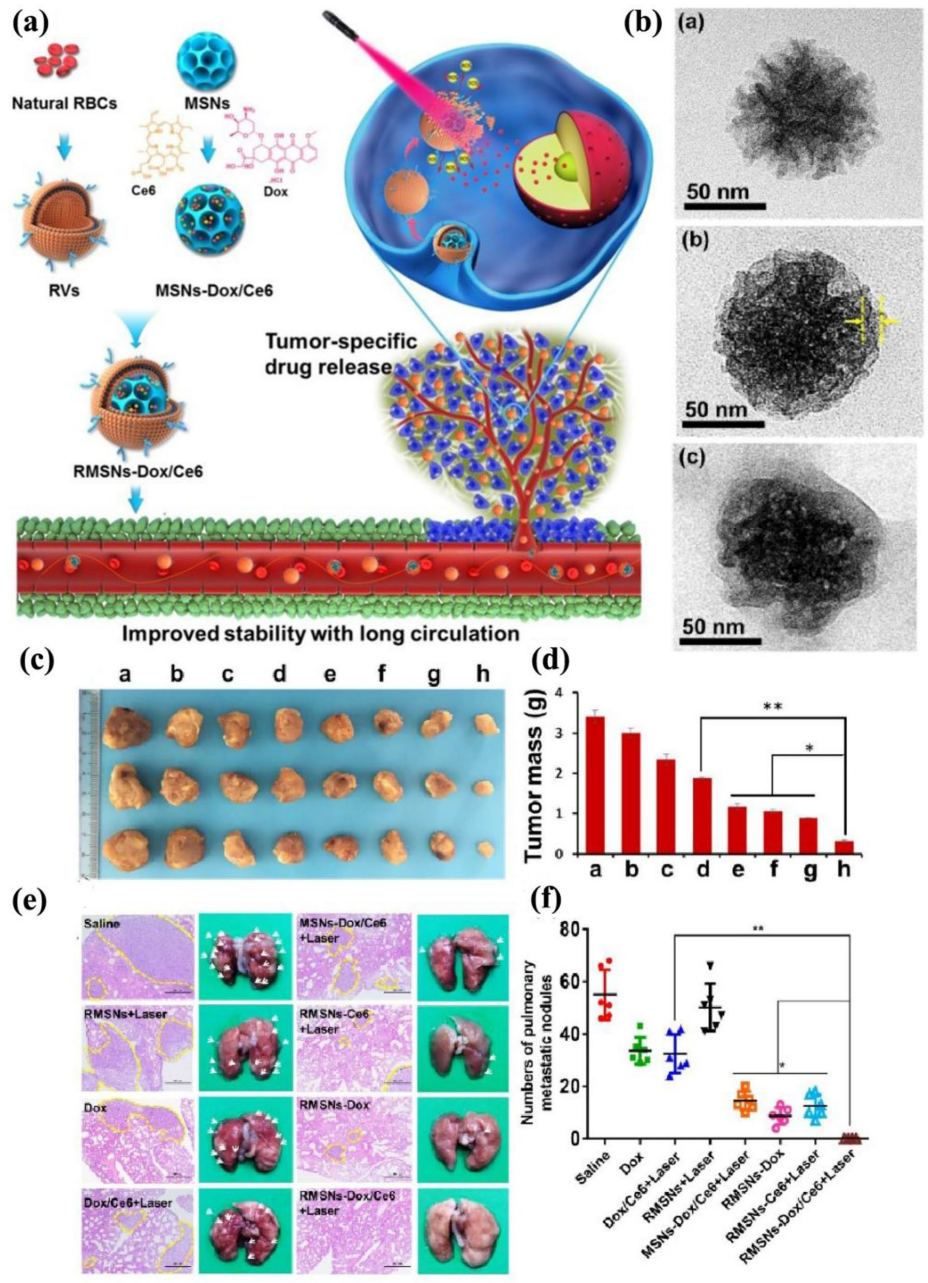
**a** The synthesis, functions, advantages, and applications of RMSNs-Dox/Ce6. **b** TEM of (**a**) MSNs-Dox/Ce6 (**b**)RMSNs-Dox/Ce6 (**c**) RMSNs-Dox/Ce6 + laser **c** Digital photos of the tumor. **d** The corresponding analysis of tumor mass. **e** Microphotos (right) and H&E stained sections (left) of lungs in different groups. The pulmonary metastases are circled with yellow dotted lines. **f** Analysis of the pulmonary metastasis nodules in different groups. Reprinted with permission from Refs [59]

Silica deposits abundantly in bone, cartilage, and other supporting tissue as an endogenous substance [52, 53]. The US FDA approved silica generally as safe in the human body, and amorphous silica was first put forward as a drug delivery carrier in 1983 [54, 55]. Since then, many drug delivery systems based on various amorphous silica have been developed rapidly [56–58]. Specifically, MSNs have aroused the robust interest of researchers since they are characterized by controllable structures and surface morphology on a nanometer scale. The mesoscopically ordered pore structure, the consequential high surface area, and the pore volume make it possible for high loading degree capacity (up to 50 wt%) and promote a controllable release of the loading drug after being modified by responsive shell materials. However, MSNs are likely to aggregate and leak into the bloodstream, which undermines their drug delivery efficacy. Su et al*.* loaded the anti-cancer drug DOX and the near-infrared photosensitizer chlorin e6 (Ce6) into MSNs, and wrapped the compound with RBCMs (Fig. 4a) [59]. Moreover, transmission electron microscope (TEM) images showed that the release of DOX in breast cancer could be controlled by the laser stimuli, which generated ROS to destroy the RBCMs shell to achieve controlled release (Fig. 4b). The digital photo and mass analysis of the tumors after 22 d treatment were shown in Fig. 4c, d, respectively. The change of the tumor size could demonstrate that MSN with Dox/Ce6 loaded (RMSN-Dox/Ce6) obtained the effects of PDT and chemotherapy synergistically to achieve the most potent anti-cancer effect under the circumstance of laser light stimulation. Moreover, as hematoxylin and eosin (H&E) staining demonstrated, laser-stimulated RMSN-Dox/Ce6 reinforced anti-metastatic efficiency dramatically (Fig. 4e). No metastatic nodules were found in the lungs, which indicates the cancer cell was limited in the tumor sites with no metastasis (Fig. 4f). This work suggested a promising potential ability of the RBCM mimetic MSNs for anti-metastasis tumor therapy, which also provided new insight into MSNs optimization.

At present, the technology for preparing INPs and RBC membranes is relatively mature, and it is entirely feasible to achieve large-scale production. To sum up, by camouflaging INPs with RBCM, the short half-life of INPs can be prolonged, and the biocompatibility of INPs can be improved, making them more suitable for drug delivery, tumor imaging, etc*.* However, the limited targeting capacity restricts the application of RBCM-INPs to achieve precise cancer therapy. The accumulation of RBCM-INPs in the tumor site mainly relies on the leaking blood vessels, while the tumors are heterogeneous and not all tumors have leaking blood vessels. Furthermore, even in different parts of the same tumor, the features of vessels are different. Under these circumstances, the use of existing drugs and RBCM-INPs delivery systems are relatively inefficient. Hence, it is necessary to confer the satisfactory active tumor-targeting ability for RBCM-INPs to attain the acceptable drugs and INPs biodistribution and therapeutic index. Currently, the active targeting of most delivery systems results from chemical modification. However, chemical modification is not suitable for RBCM-INPs, which may affect the biodistribution of surface proteins and even damage the integrity of the membrane, which may consequently destroy the membrane’s function. Fang et al*.* demonstrated a lipid-protein insertion method to functionalize RBCM, inserting targeting ligands to endow RBCM with cancer-targeting abilities [60]. Apart from cell membrane modification, the technology of fusing RBCM with other cell membranes to obtain cancer-targeting ligands has been invented, which will be thoroughly introduced in our review.

## Cancer cell membrane-camouflaged inorganic nanoparticles (CCM-INPs)

Compared with blood cells, cancer cells can evade immune surveillance and target homologous cells due to specific proteins and receptors on their surfaces. Cancer cell membranes (CCMs) retain the cell adhesion molecules from the surface of source cells, including selectins, cadherins, immunoglobulin superfamily, integrins, and lymphocyte homing receptors [61]. The self-markers and self-recognition molecules enable CCM-INPs to escape immune clearance and be hidden in the bloodstream. Therefore, coating CCM can extend their circulation time in the body, and greatly improve the homologous targeting ability of INPs [62].

**Fig. 5:**
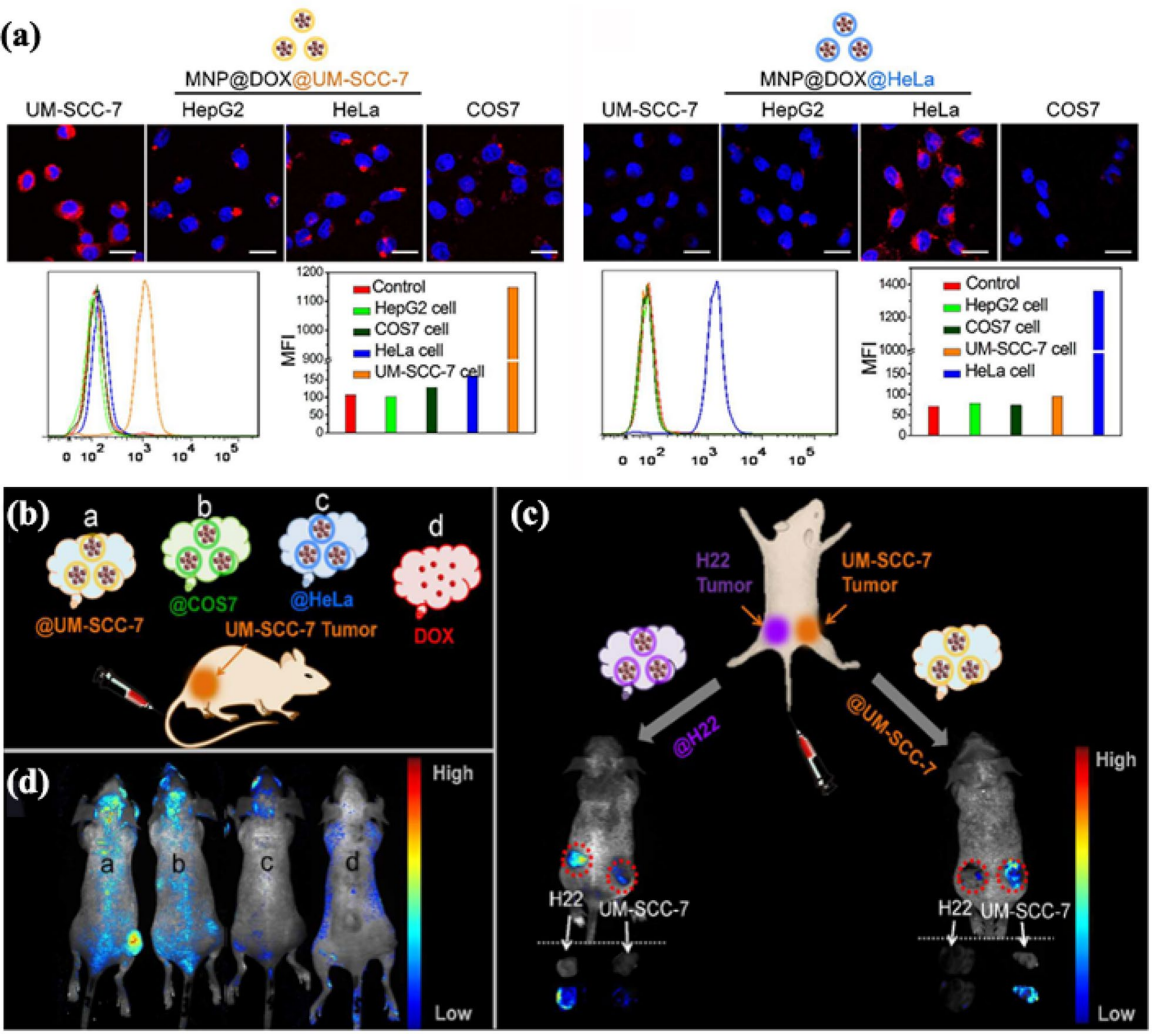
**a** CLSM images and flow cytometric profiles of four cell lines including UM-SCC-7, HepG2, HeLa and COS7 cells upon 2 h co-incubation with MNP@DOX@UM-SCC-7 (left) and MNP@DOX@HeLa (right). **b** Schematic illustration of UM-SCC-7 tumor-bearing mouse model treated with DOX and various MNP@DOX@CCMs. **c** In vivo fluorescence images of MNP@DOX@CCMs (**a**: @UM-SCC-7, **b**: @COS7, **c**: @HeLa, d: DOX) with an equivalent DOX dosage. **d** Illustration of H22 and UM-SCC-7 dual-tumor bearing mouse model. Reprinted with permission from Refs [63]

Zhu et al*.* developed magnetic iron oxide-based NPs (MNPs) loaded with DOX and camouflaged them with different types of CCMs derived from homotypic UM-SCC-7 squamous carcinoma cells, COS7 monkey kidney cells, and HeLa cervical cancer cells [63]. As depicted in Fig. 5a, the results of confocal laser scanning microscopy (CLSM) images and flow cytometric profiles show that the using CCMs for camouflaging MNPs expresses a remarkable ability to self-recognition of origin cells and good immune escape in vitro. More significantly, even in competition with other xenogeneic tumors, MNP@CCMs also show highly selective targeting for homologous tumors in vivo. Firstly, injected intravenously in the right hind limb of UM-SCC-7 tumor-bearing mice with DOX alone or different MNP@DOX@CCMs, as validated in Fig. 5b (a: @UM-SCC-7; b: @COS7; c: @HeLa; d: only DOX). At 24 h, the living fluorescence images in vivo were performed (Fig. 5c). It exhibited the specificity of homologous membranes targeting, as only MNP@DOX wrapped up in the UM-SCC-7 cell membranes displayed much stronger fluorescence than other groups, indicating the efficient accumulation of MNP@DOX@UM-SCC-7 towards tumors (Fig. 5d). After that, the second mouse model that carried two different types of tumors was established to further verify the homologous targeting effect (H22 on the left hind limb and UM-SCC-7 tumor on the right). MNP@DOX@H22 was injected intravenously into the second mouse model. Compared with the fluorescence intensity of UM-SCC-7 tumors, in vivo and in vitro fluorescence imaging clearly showed the accumulation of NPs in H22 tumors. MNP@DOX@UM-SCC-7 exhibited the strongest affinity for UM-SCC-7 tumors. The specific ability of self-targeting that home to the homotypic tumor in vivo of CCM-INPs has been proved. At the same time, this “homing” targeting ability makes drug-loaded CCM-INPs show excellent tumor therapeutic effects in vivo. This bionic strategy showed great potential to accurately treat and diagnose diverse tumors only by modulating the source of CCM on the NPs accordingly.

INPs are a kind of nanomaterials that have entered the view of researchers for decades and can be applied to PTT. Nowadays, researchers are no longer limited to a single PTT but are loading different components to achieve combined therapy. Sun et al*.* reported a core–shell nanosystem with DOX-loaded gold nanocages (AuNs) as inner cores and 4T1 CCM as outer shells (CCM@AuNs) [64]. The CCMs@AuNs perfectly utilized the CCMs with homotypic targeting and combined with AuNs as the photothermal agent to realize the selective targeting of the homotypic tumor cells, hyperthermia-triggered drug release under the near-infrared laser irradiation, and the combination of chemo/photothermal therapy. Furthermore, Sun et al*.* also established cancer cell membrane-camouflaged gold nanorods (GNR@Mem) possessing excellent photothermal conversion ability within the NIR-II window and radiosensitizing ability under X-ray irradiation [65]. Therefore, GNR@Mem can serve as a promising platform for cancer photothermal therapy and radiotherapy in vitro and in vivo*.* Nowadays, with the maturity of loading component technology, adding functional components to achieve multi-modal therapy in INPs would become a trend in the future.

**Fig. 6:**
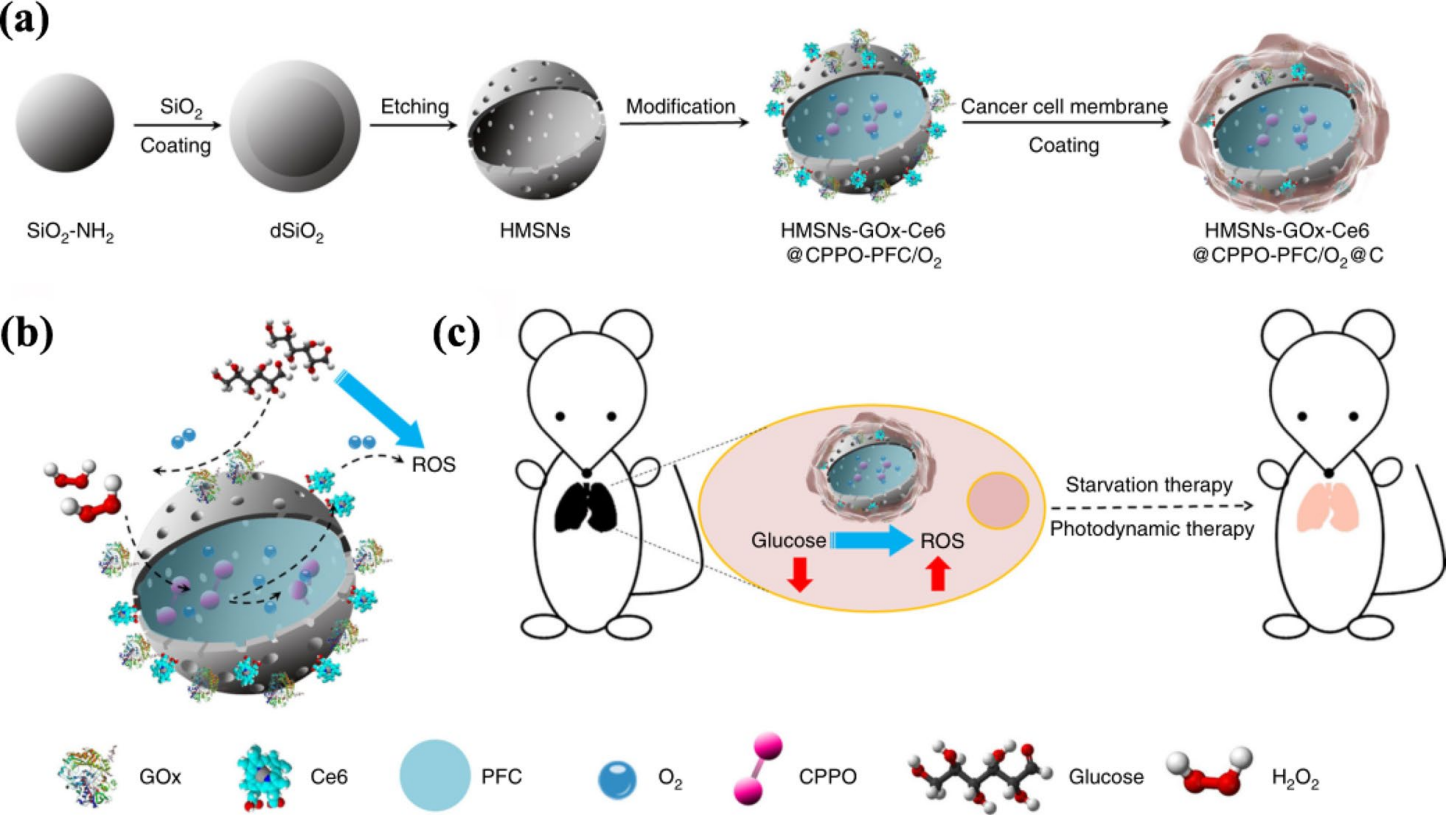
**a** Schematic illustrations of the process for synthesizing the biomimetic nanoreactor. **b** ROS generation based on CRET with glucose consumption with no light excitation. **c** Synergetic photodynamic-starvation therapy for metastasis. Reprinted with permission from Refs [66]

With the in-depth exploration of cancer metabolism, a new cancer treatment method named starvation therapy was developed. By restricting or depriving the survival demand conditions of the tumor cells, the purpose of “starving” the tumor cells is realized. Ge et al*.* designed a chemiluminescence resonance energy transfer (CRET)-based biomimetic nanoreactor (bio-NR) to realize combined photodynamic-starvation therapy against tumor metastasis [66]. The assembly process of the materials is demonstrated in Fig. 6a. The Ce6 and glucose oxidase (GOx) were first loaded into the hollow mesoporous silica nanoparticles (HMSNs) and modified co-encapsulating bis[2,4,5-trichloro-6-(pentyloxycarbonyl)phe-nyl] oxalate (CPPO) and perfluorohexane (PFC) on the surface, followed by coating CCM on the surface. In this bio-NR, the Ce6 is excited depending on the energy from the reaction between CPPO and intracellular H_2_O_2_ originating from the tumor in a hypermetabolic state to generate ROS for cancer therapy. Meanwhile, the GOx on the HMSNs converts glucose to H_2_O_2_ through a series of reactions at the tumor site, which could not only cut off the energy supply to achieve the purpose of starvation treatment but also provide H_2_O_2_ to enhance the ability to generate ROS. The details of using synergetic PDT and starvation therapy via CRET against cancer metastasis are illustrated in Fig. 6b, c. Furthermore, PFC can improve the hypoxic tumor microenvironment through the O_2_ carrying and accelerate the glucose oxidation to promote ROS generation. Moreover, the CCMs coating makes bio-NR have an excellent targeting ability. This work showed that the strategy of PDT combined with starvation therapy had a significant killing effect on cancer cells and effectively prevented the recurrence of cancer.

In recent years, cancer immunotherapy has been considered as one of the most promising strategies to treat cancer. CCMs have a series of tumor-associated antigens (TAAs) on the surface, which trigger tumor-specific immune responses in vivo and are a good choice to be used in cancer vaccines. Fang et al*.* designed a biomimetic nanovaccine coated with Melanoma cell membrane (B16-F10) to achieve tumor-specific immune responses [67]. They utilized TAAs from CCMs to target homologous tumors and antigen-presenting cells (APCs). Consequently, composite NPs could stimulate superior immune responses to fight against cancer cells. However, an effective immune stimulation requires not only tumor antigens but also adjuvants. Nano-vaccines, in the absence of immune adjuvants, are insufficient to induce dendritic cell maturation. To overcome this disadvantage, Kroll et al*.* developed B16-F10 membrane-wrapped INPs creatively loaded with CpG, a nucleic acid-based immunological adjuvant widely known to trigger APCs maturation [68]. The results indicated that it could significantly enhance the degree of immune activation after adding adjuvants. Thus, the combination of CCM-INPs and adjuvants is an advanced strategy to elicit intense anti-tumor responses.

From the introduction to the above of CCM and INPs, the combination of CCM and INPs is an efficient approach to solving the clinical problems of INPs. Next, the common INPs coated cancer cell membranes (Table 1) are summarized to provide some new ideas for the way forward to clinical applications.

**Table 1:** Summary of common INPs, coated cancer cell membranes, loaded cargos, and purposes

Category	Core INP Material	Cancer Membrane Source	Cargo Loaded	Purpose	Ref
Metal nanomaterials	Gold nanocages	4T1	Doxorubicin	PTT; Hyperthermia-triggered drug release	[64]
Rare-earth-doped nanomaterials	Ln-doped upconversion Nanocrystal	MDA-MB-435;CAL27; HCT116;DU145	Null	FL imaging	[69]
Sulfide nanomaterials	Hollow copper sulfide	B16-F10^a^	Doxorubicin	Drug delivery	[70]
Oxide nanomaterials	Mesoporous silica	4T1	Glucose oxidase	Immunotherapy; Starvation therapy	[71]
	Mesoporous silica	LNCaP-AI	Doxorubicin; Calcium carbonate	Drug delivery; Ph-sensitive release	[72]
	Mesoporous silica	MCF-7	Doxorubicin; Parp inhibitor mefuparib hydrochloride	Drug delivery	[73]
	Hollow manganese Dioxide	B16-F10	Chlorin e6; Glucose oxidase	PDT; Starvation therapy	[74]
	Iron oxide	UM-SCC-7;HeLa; HepG2; COS7	Doxorubicin	Drug delivery	[63]
	Superparamagnetic iron oxide	SMMC-7721	Chlorin e6	PDT; MRI imaging	[75]

^a^Membranes were mixed with red blood cell membranes before coating

Although many of the successes described that the CCM-INPs systems are very exciting, many challenges still need to be solved before these technologies can be commercialized. The potential problem is that people may be worried about injecting substances derived from cancer cells into the body, especially the patients who are at risk of certain types of cancer and want to accept this technology as a preventive vaccine. Therefore, strict tests and procedures must be established to ensure a pure CCM (without any internal components of the cancer cell) and not containing any molecules that may promote cancer growth. One of the biggest attractions of coating nanoparticles with cell membranes is the ability to provide personalized treatment. However, whether it is feasible to create CCM-INPs for each patient is an important question. It may be a key factor limiting the commercial scale of nanoparticles. Although it may be difficult to achieve clinical transformation, the opportunity to eradicate even one cancer through treatment or vaccine will continue to promote many research approaches to CCM-INPs.

## Mesenchymal stem cell membrane-coated inorganic nanoparticles (MSC-INPs)

The stem cell is a series of multipotent progenitor cells that can self-replicate and differentiate into multiple cells [76]. Particularly, mesenchymal stem cell (MSC) is a critical member of the stem cell family, which has been attracting widespread attention in the past decades for their good immune compatibility, tumor affinity, and easy expansion in vitro [77]. MSC can be extracted from different tissues of the human body, including bone marrow, umbilical cord, and adipose tissue [78, 79]. It is worth mentioning that MSC has a great tendency toward the developing tumors ascribed to MSC tumor-homing effects [80]. MSC homing is the process by which MSC migrates to the targeted tissue and colonizes under the action of various biotic factors. As for cancer, persistent wound healing is a definite process during its development. The over-expressed adhesion molecules, chemokines, and growth factors, which are the integral components of tumor stroma, induce MSC to migrate to tumor sites actively [32]. Meanwhile, these specific signaling molecules rich in the tumor microenvironment can bind to the corresponding MSC membrane surface proteins, all these factors contributing to MSC tumor-homing behavior. Therefore, the MSC membrane is an ideal potential carrier for establishing a drug delivery system that promises to minimize adverse off-target effects for cancer treatment.

**Fig. 7:**
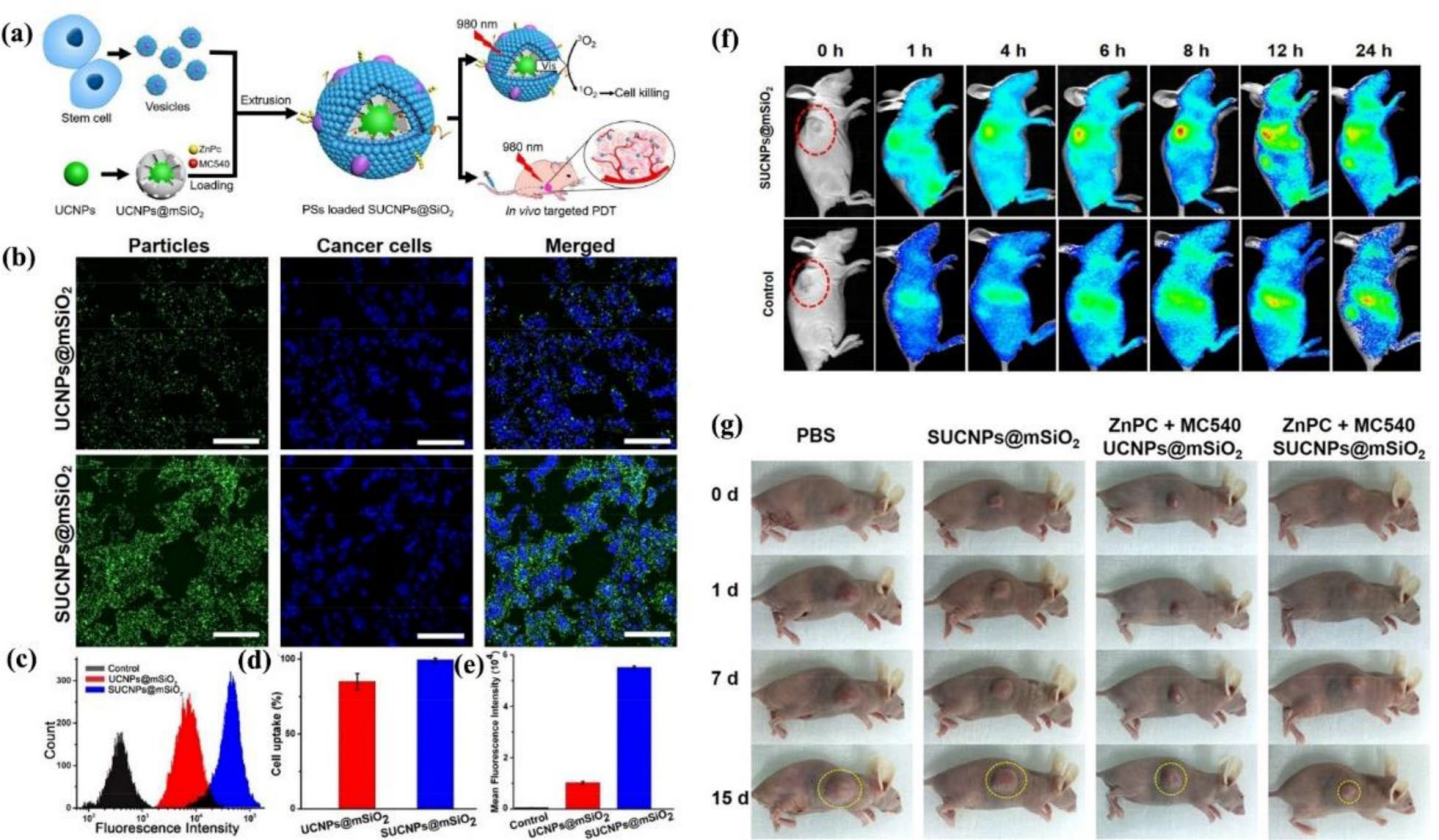
**a** The fabrication process of SUCNPs@mSiO_2_ and their mechanism in photodynamic therapy. **b** Enhanced in vitro cancer cell accumulation of SUCNPs@mSiO_2_. CLSM images demonstrate the tumor cell binding of UCNPs@mSiO_2_ and SUCNPs@mSiO_2_. All scale bars are 200 nm. **c** Quantitative analysis of HeLa cells by flow cytometry. **d** Percentages of HeLa cells with increased fluorescence in **(c)**. **e** Quantification of the mean fluorescence intensity of HeLa cells in **(c)**. **f** In vivo fluorescence images of mice at 1, 2, 4, 8, 12, and 24 h after injection of Cy7-SUCNPs@mSiO_2._ The red circles indicate the tumor sites. **g** Photographs of mice show the tumor size change after various treatments at different time points. Reprinted with permission from Refs [81]

In 2016, He et al*.* combined the MSC membrane with dual photosensitizers-loaded mesoporous silica through a mechanical extrusion method (Fig. 7a) [81]. CLSM images of the UCNPs@mSiO_2_ and MSC membrane-coated UCNPs@mSiO_2_ (SUCNPs@mSiO_2_) in different circumstances in vitro are shown in Fig. 7b. It demonstrates that when incubating the UCNPs@mSiO_2_ and SUCNPs@mSiO_2_ with cancer cells, the SUCNPs@mSiO_2_ showed higher fluorescence intensity, which indicated that the MSC membrane is reliable for tumor affinity. Fluorescence intensity is quantified by the flow cytometry in Fig. 7c–e. For in vivo tumor targeting evaluation of SUCNPs@mSiO_2_, He et al*.* injected Cy7-SUCNPs@mSiO_2_ into the tail vein of mice and used Cy7-UCNPs@mSiO_2_ with the same amount as a control. As shown in Fig. 7f, the fluorescence intensity of Cy7-SUCNPs@mSiO_2_ at the tumor site was more than the control group, which indicated the tumor homing effect of SUCNPs@mSiO_2_ endowed by the MSC membrane. Collectively, MSC membrane camouflaging is a reasonable pathway to improve the efficacy of anti-cancer therapy in vivo. This precocious work verified that this STM-camouflaged UCNPs-based nanoplatform had a promising ability of deep-tissue PDT for cancer treatment. Collectively, MSC membrane camouflaging is a reasonable pathway to improve the efficacy of tumor affinity in vitro and tumor homing in vivo. As illustrated in Fig. 7g, compared with the tumors in the control groups, tumors in the PS-loaded SUCNPs@mSiO_2_-injected mice were remarkably inhibited. This pioneering work verified that this STM-camouflaged UCNPs-based nanoplatform had a promising ability of deep-tissue PDT for cancer treatment. MSCs in this work has advantages of easy cultivation and amplification in vitro*,* low immunogenicity, and low ethical controversy. More investigations can be carried out by exploiting the merits of other stem cell membranes to endow MSC-INPs with more biological functions.

**Fig. 8:**
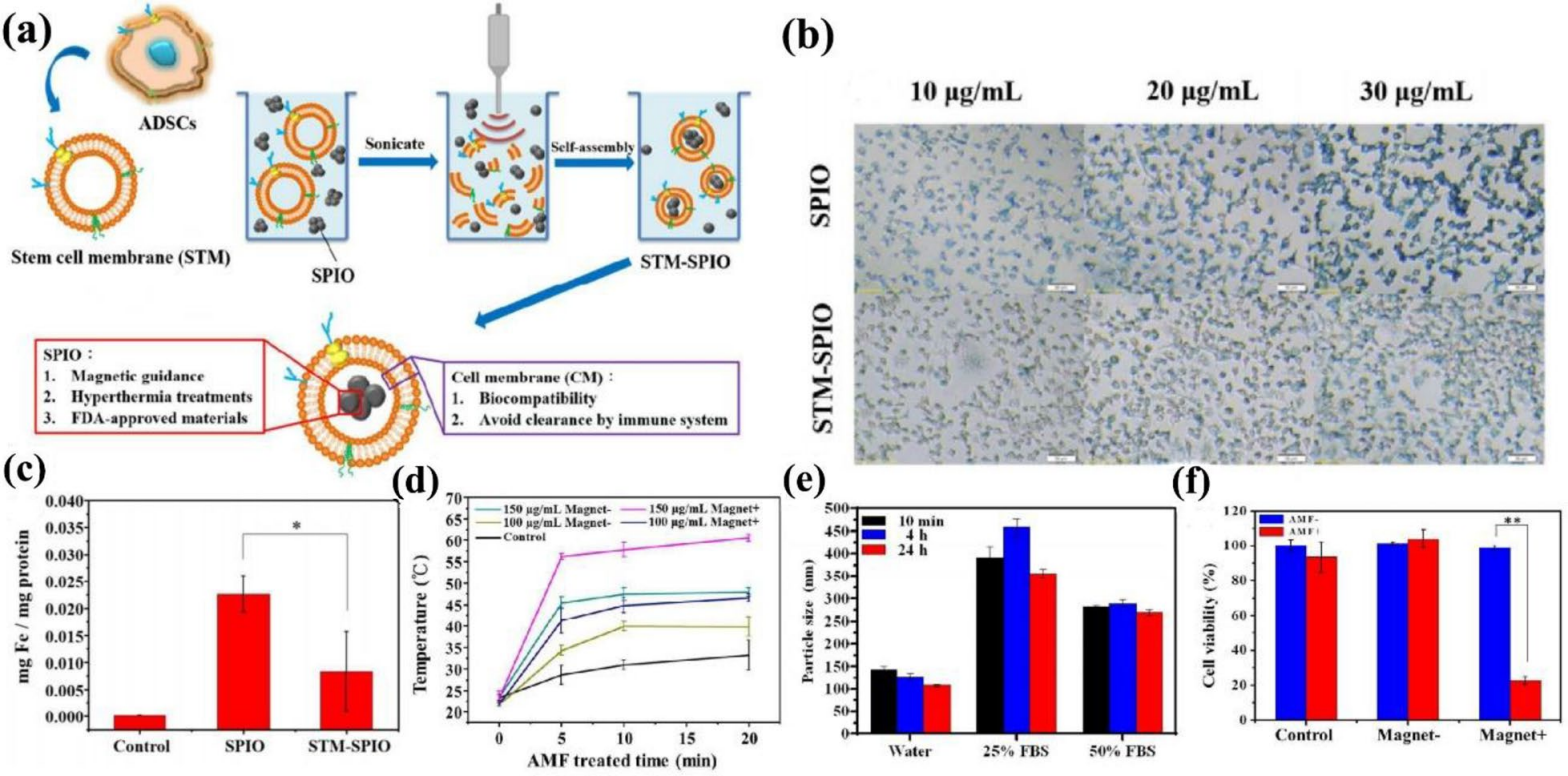
**a** Schematic representation of STM-SPIO preparation procedure. **b** Macrophage uptake of SPIO and STM-SPIO of three different concentrations. **c** Quantitative measurement of intracellular Fe level in the macrophages by using inductively coupled plasma mass spectrometry (ICP-MS). **d** Effect of alternating magnetic field (AMF) treatment on prostate cancer cells solutions. **e** The particle size of MSC membrane-camouflaged superparamagnetic iron oxide NPs incubated with water, 25% and 50% FBS-containing DMEM at 30 min, 4 h, and 24 h, respectively. **f** Viability assay of prostate cancer cells. Reprinted with permission from Refs [82]

In 2017, Chang et al*.* designed a biomimetic nanoplatform of MSC membrane-camouflaged superparamagnetic iron oxide NPs (STM-SPIO), which achieved self-assembling through sonication. (Fig. 8a) [82]. After being co-incubated with DI H2O and cell culture medium for 10 min, 4 h, and 24 h, respectively, the particle size of the biomimetic nanoplatform did not change significantly, which signified the adequate colloidal stability for further therapeutic use. (Fig. 8e) Macrophages were incubated with various concentrations (10, 20, and 30 μg/mL) of SPIO (Top) and STM-SPIO (Bottom). STM-SPIO showed lower macrophages uptake than the bare SPIO (Fig. 8b c), which implied better biocompatibility and a longer circulation time of STM-SPIO. As shown in Fig. 8d, when exposed to an alternating magnetic field (AMF) in vitro, the 150 μg/mL Magnetic^+^ membrane-coated group achieved the highest temperature, representing the most effective PTT efficacy. The prostate cancer cells were killed rapidly via the magnetocaloric effect (Fig. 8f). In conclusion, the multifunctional nanoplatform holds immense prospects for extensive biomedical applications for active targeting drug delivery systems, magnetic resonance imaging, and magnetic hyperthermia therapy in the future. However, this work only verified the efficient anti-cancer function of this biomimetic nanoplatform in vitro. It is also interesting to identify their biosafety and explore other biomedical applications, including tissue repair, antibacterial, and so on.

However, the source of MSCs is scarce, greatly limiting the development of MSCM-INPs. Meanwhile, it costs a great deal of money and medical resources to acquire MSCM, which also restricts the clinical applications of MSCM-INPs compared with other types of CMCINPs.

## White blood cell membrane-camouflaged inorganic nanoparticles (WBCM-INPs)

White blood cell (WBC) exists widely in blood and lymph with the ability to migrate actively, which mainly exerts immune defense and regulation functions in vivo [100]. According to its granularity and shape difference, WBC is divided into five major types, including monocytes/macrophages, neutrophils, eosinophils, basophils, and lymph cells [101]. WBC expresses abundant signal and adhesion molecules on its surface, which makes the WBCM-INPs partly preserve the functions of the WBC. Therefore, WBCM-INPs have a wide prospect of application in TDDS [83, 84].

**Fig. 9:**
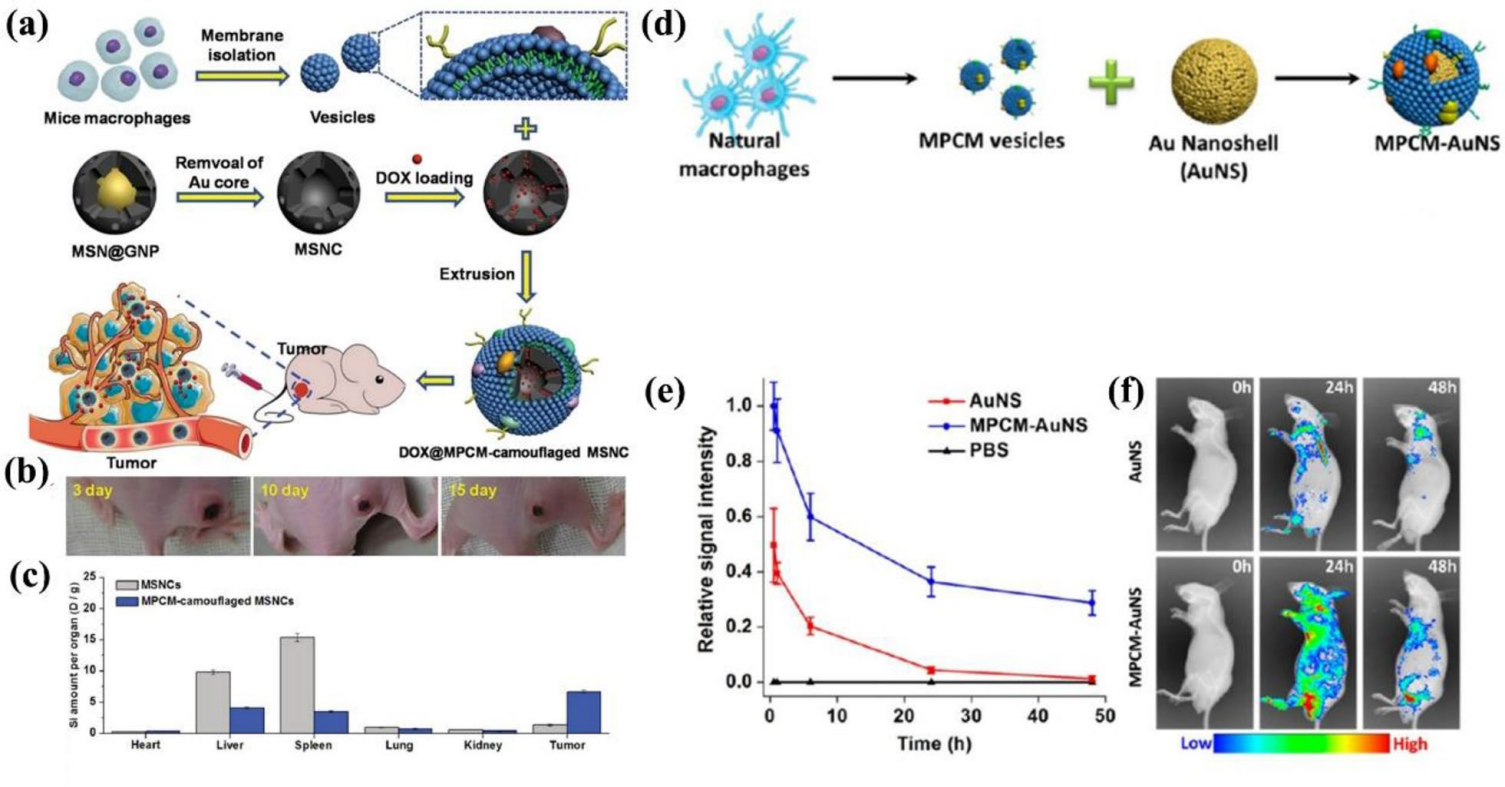
**a** The preparation process of the MPCM-camouflaged MSNCs and application for subsequent in vivo cancer therapy. **b** The tumor growth in mice treated with DOX@MPCM-camouflaged MSNCs. **c** The amount distribution of NPs in tumors and different organs. **d** The brief synthesis process of MPCM-AuNS. **e** The relative signal intensity of AuNS, MPCM-AuNS, and PBS after intravenous injection. **f** The images of mice bearing 4T1 tumor injected AuNS and MPCM-AuNS under the fluorescence time-lapse. Reprinted with permission from Refs [85, 86]

Long-term chronic inflammation is a significant symbol of the malignant tissue, and therefore macrophages and neutrophils can target and get enriched in tumor sites [83]. These characteristics of WBC provide creative ideas to design a carrier for tumor targeting therapy. MSNs are always widely used as a drug delivery platform due to their porous structure and large specific surface area, but the bare MSNs cannot target the specific tumor site. Xuan et al*.* coated the macrophage membrane (MPCM) on DOX-loaded MSNs and proved the MPCM-INPs carrying drugs could target and get enriched in the tumor site to kill the tumor tissue after injection (Fig. 9a) [85]. The results showed that the MPCM-camouflaged MSNs could cause higher toxicity to 4T1 breast cancer cells in vitro than free DOX and DOX-loaded MSNs. Figure 9b showed the reduced tumor volume after being treated with MPCM-camouflaged NPs in mice tumor-bearing models over time (3, 10, and 15 d). Due to the excellent targeting ability of the MPCM, the MPCM-camouflaged MSNs had a higher accumulation in the tumor than bare MSNs, and a lower accumulation in organs such as liver and spleen, which indicated that MPCM-camouflaged MSNs also had the ability of immune escape (Fig. 9c). This work suggested that the inflammatory environment can serve as a targeting area to increase the concentration of CMCINPs at tumor sites, which takes advantage of the interaction between WBCM and the unique tumor microenvironment. However, the specific mechanism of interaction needs further study. As an example, the specific molecules on WBCM that can interact with the tumor microenvironment should be confirmed. Meanwhile, it should be noted that the interaction is also affected by the type and the stage of tumors.

In addition to the ability of targeting, immune escape and prolonged circulation time are also significant. Since the gold NPs can convert light to thermal energy, inducing local temperature increase in tumor sites, they can be used for cancer PTT [40]. However, the half-life of circulation is short due to the intense clearance by the immune system. Xuan et al*.* reported the macrophage cell membrane-camouflaged Au nanoshells (MPCM-AuNSs) through a top-down assembly method to prolong the blood circulation time (Fig. 9d) [86]. First, Xuan et al*.* coated the mesoporous silica onto the gold core, and then the composite particles were camouflaged with a macrophage membrane. Meanwhile, they loaded dye on the silica to achieve the PTT and imaging integration simultaneously. As shown in Fig. 9e, through the detection of the relative signal intensity, they found that the MPCM-AuNSs had a longer circulation time than bare AuNSs. And in the in vivo experiments, lower clearance of NPs achieved only when AuNSs were camouflaged with MPCM (Fig. 9f). The longer circulation time expressed better effects of killing tumors and fewer adverse effects. Then, they treated the nude mice bearing the 4T1 tumor in different therapeutic conditions, and the tumor volume was much smaller in the mice injected with the MPCM-AuNSs than in other control groups. In summary, MPCM-camouflaged AuNSs retain the natural properties of MPCM, which improved the PTT efficacy modulated by AuNSs and other metal INPs. It is worth mentioning that the WBCs in this work utilized natural macrophages. However, to the best of our knowledge, the natural macrophages can be polarized into M1 or M2 types under specific stimulation. Since different types of macrophages have various influences on the process of tumor progression, more research could focus on camouflaging INPs with various types of macrophages to investigate their anti-cancer and biological functions.

**Fig. 10:**
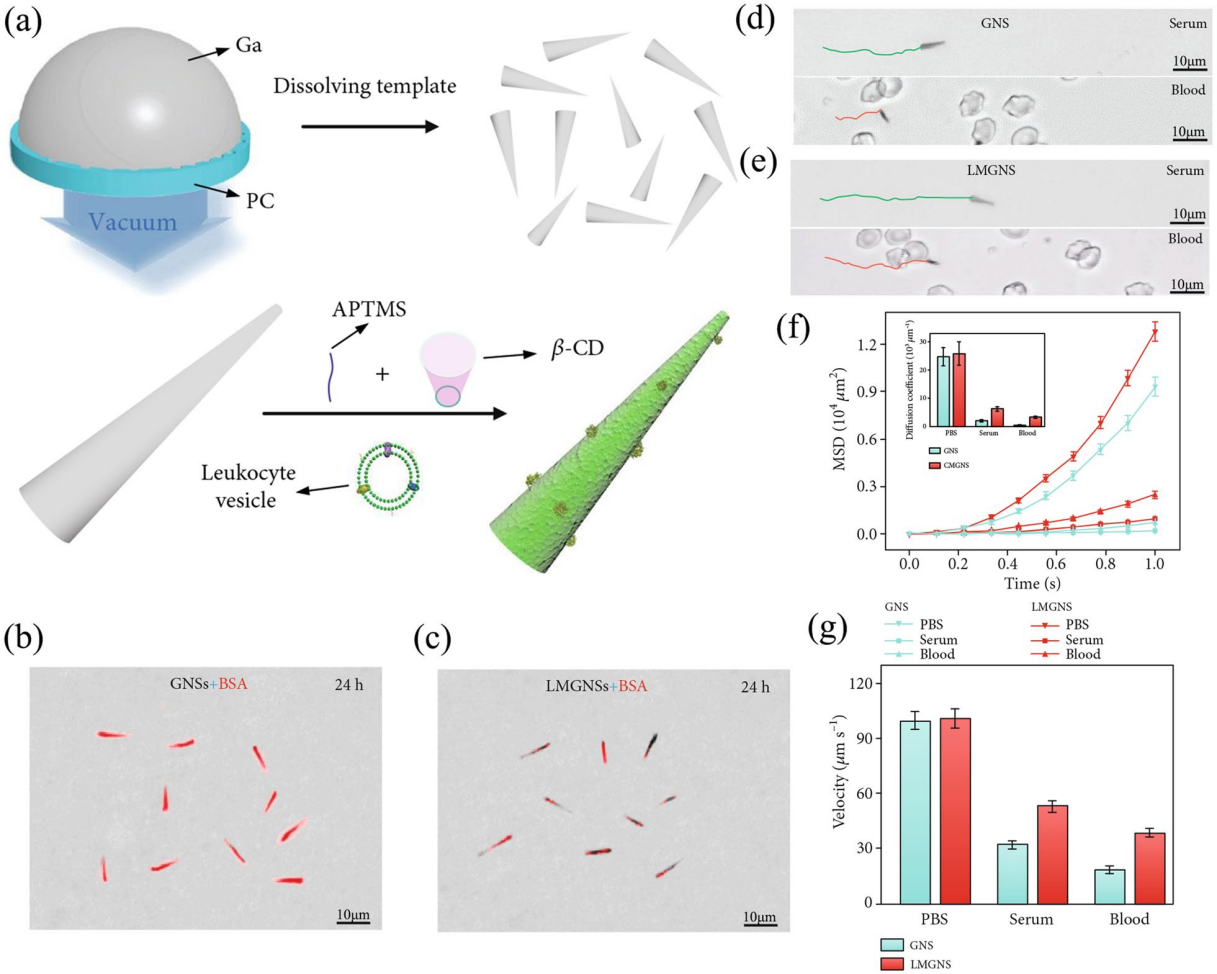
**a** Schematic illustration of the fabrication process of LMGNSs. **b** The CLSM images of GNSs after co-cultured with Rhodamine-labeled BSA for 24 h. **c** The CLSM images of LMGNSs after co-cultured with Rhodamine-labeled BSA for 24 h. **d** The movement distance of GNSs in the blood and serum. **e** The movement distance of LMGNSs in the blood and serum. **f** The velocity of GNSs and LMGNSs in PBS, serum, and blood media. **g** The mean-squared displacement (MSD) and diffusion coefficient of the GNSs and LMGNSs in the different solutions. Reprinted with permission from Refs [91]

Nanoswimmer can translate various types of energy to mechanical movement, and it is often used for biosensing, drug delivery, and PTT [87]. As a kind of nanoswimmers, liquid metal has excellent biocompatibility, and the movement of liquid metal can be regulated through the alternating frequency and voltage of the ultrasonic field, and therefore this nanoswimmer is given the ability of autonomous motion [88, 89]. However, when the bare gallium nanoswimmer (GNS) enters the circulatory system, they will be affected by the “biofouling effect” vulnerably, which will enhance the effects of immune clearance and viscous resistance [90]. It was reported that Wang et al*.* synthesized the leukocyte membrane-camouflaged gallium nanoswimmers (LMGNSs) (Fig. 10a) [91]. It showed a fascinating capability of the anti-biofouling after camouflaging with the WBC membrane. As shown in Fig. 10b, c, after incubation with Rhodamine-BSA, the fluorescence signal of GNSs was higher than LMGNSs. It proved that the LMGNSs were not susceptible to protein adsorption. To evaluate the motion behavior of the nanoswimmer, Wang et al*.* tested the movement distance of GNSs and LMGNSs (Fig. 10d, e). The LMGNSs moved with a longer distance than GNSs in serum and blood, representing a better anti-biofouling effect. And through the coefficient detection of the velocity and diffusion (Fig. 10f, g), the LMGNSs showed a better locomotivity in the biological media. Meanwhile, this nanoswimmer certainly exhibited the ability of photothermal effect, making it possible for PTT. This biomimetic nanoplatform integrated the abilities of active driven motion, imaging, cancer cell targeting, drug delivery, antibio-fouling, and PTT altogether, making this nanoswimmer a next-generation theranostics platform. The leukocyte membrane here can act as a protective measure to prevent the directionally moving nanoswimmer from being affected by the biofouling effect, thus prolonging the motion cycle of the nanoswimmer. With the rise of remote-controlled INPs for cancer diagnosis and therapy, this WBCM-camouflaging technology can be applied extensively to prolong the duration of action of INPs. However, intricate motion of INPs, such as rotation, bounce, might be affected by the coated WBCM.

**Fig. 11:**
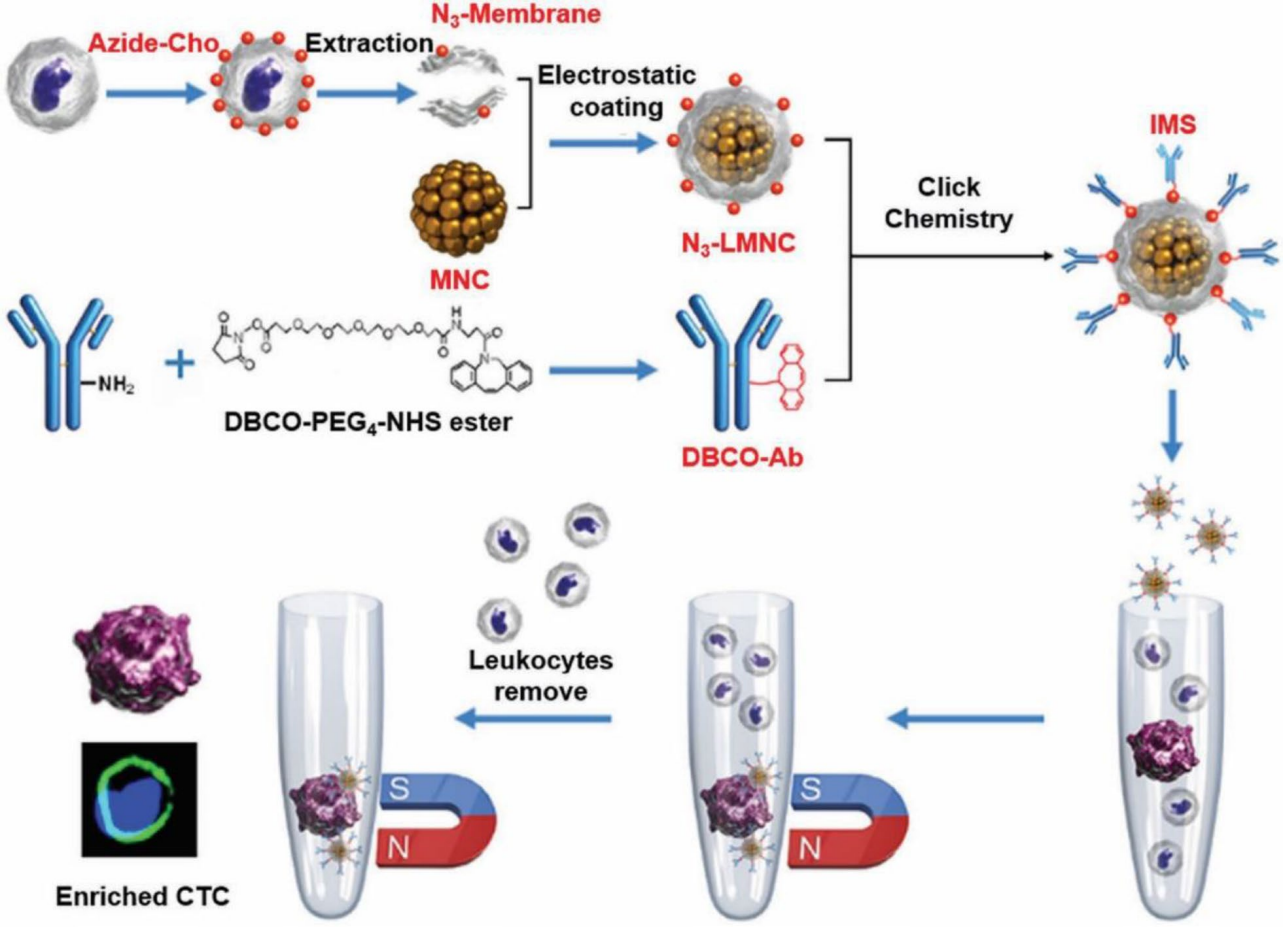
Construction of IMS and the procedure of CTC enrichment.Reprinted with permission from Refs [96]

In terms of the detection of cancer cells, WBCM-INPs exhibit a creative application due to the combination of targeting and immune escape. Circulating tumor cells (CTCs) is considered a crucial step in forming metastases, which determines that screening and detecting CTCs is important for early cancer diagnosis, treatment monitoring, and prognostic evaluation [92, 93]. However, the amount of CTCs in the blood is very low among the surrounding background cells, which makes it hard to collect them [94]. Xiong et al*.* developed biomimetic immuno-magnetosomes (IMSs) to enrich CTCs. (Fig. 11) The leukocyte membrane was modified with azide (N_3_), paving the way for subsequent Ab decoration by chemical modification, which endows the leukocyte membrane with efficient CTCs recognition. Then the MNCs were camouflaged by the pre-engineered leukocyte membrane to form IMSs. IMSs would be repelled when encountered a leukocyte in the bloodstream because of its homology [95]. Therefore, unspecific leukocyte absorption would be suppressed to a large extent. Surprisingly, the results showed that about 90% of the CTCs could be captured from the peripheral blood in a short period with an undetectable leukocyte background, which firmly verified IMSs are superior candidates for CTCs recognition and managing cancer therapy. This WBCM-camouflaging technology significantly increases the ability of INPs to detect specific circulatory morbid substances in terms of sensitivity and degree of purity, overcoming the dilemma of false positive and false negative results caused by background interference.

WBCM-INPs are no doubt an emerging and effective cancer therapy nanoplatform. The INPs inherit the function of the WBC stably, like inflammation sites aggregation and immune escape, which can overcome the limitations of the INPs when applying for tumor therapy. Moreover, compared to the maturely established RBCM-INPs that have been studied a lot, the WBCM-INPs are more functional because the WBC plays a relatively complex role in the human body and has complicated surface molecules. And the WBCM-INPs also inherit the transendothelial migration ability from WBC. Despite the advantages above, the source of the WBC is still a challenge, and the immune rejection should be reckoned with when choosing cell lines from donors. The application of patients' autologous WBC needs to consider the sufficient amount of WBC. It is worth mentioning that if choosing to use the tumor cell line as the source of the cell membrane, it is a necessity to abandon the carcinogenicity of its nucleic acid.

## Platelet membrane-camouflaged inorganic nanoparticles (PLTM-INPs)

Platelets are a kind of akaryotes released from the cytosol of megakaryocytes in bone marrow hematopoietic tissue and have a direct effect on thrombosis. Uncontrolled platelet activation can lead to some chronic inflammations, including atherosclerotic thrombosis and even inflammation in cancer [97]. Besides, plenty of research showed that platelets play an essential role in the pathogenesis and progression of malignant tumors. Recently, a significant cross-communication of tumor cells and platelets was found [98]. Cancer cells can “educate” platelets by affecting their RNA profiles, making a difference in the number of circulating platelets, and changing the state of platelet activation. Meanwhile, “educated” platelets can produce excess activators, including cyclic uptake and platelet-specific biomolecules, which are released from platelets upon activation and promote the development of malignant tumors. The process of primary tumor-induced platelet production, aggregation, and activation contribute to the prethrombotic state in the blood. Platelet activation is critical for tumor growth and metastatic outbreaks. Moreover, some crucial components in the tumor microenvironment, such as vascular endothelial growth factors, platelet-derived factors, and transforming growth factor β, can promote the development of malignant tumors [99]. CTC can contact, activate, and be promoted to proliferate by platelets. Compared with RBCM, platelet membrane (PLTM) encapsulation has the advantage of targeting inflammation and tumor sites. Researchers have utilized this characteristic to transport drugs and INPs to tumor sites [100]. There are two mechanisms of targeted therapy with platelet encapsulated NPs-passive targeting and active targeting [101]. Passive targeting is based on the surface ligands of platelet membrane-camouflaged NPs (PNPs) that have autologous antigens derived from PLTM, such as CD47, which helps PNPs escape the immune system elimination and deliver more drugs to the inflammation sites. In terms of active targeting, a variety of receptors on the surface of PNPs can also interact directly with specific components of malignant tissues. For example, the cell adhesion molecule P-selectin glycoprotein ligand-1 (PSGL-1) can specifically recognize the CD44 that is overexpressed on the surface of the tumor cell membrane. All these interactions endow PNPs with the ability to target tumors actively.

**Fig. 12:**
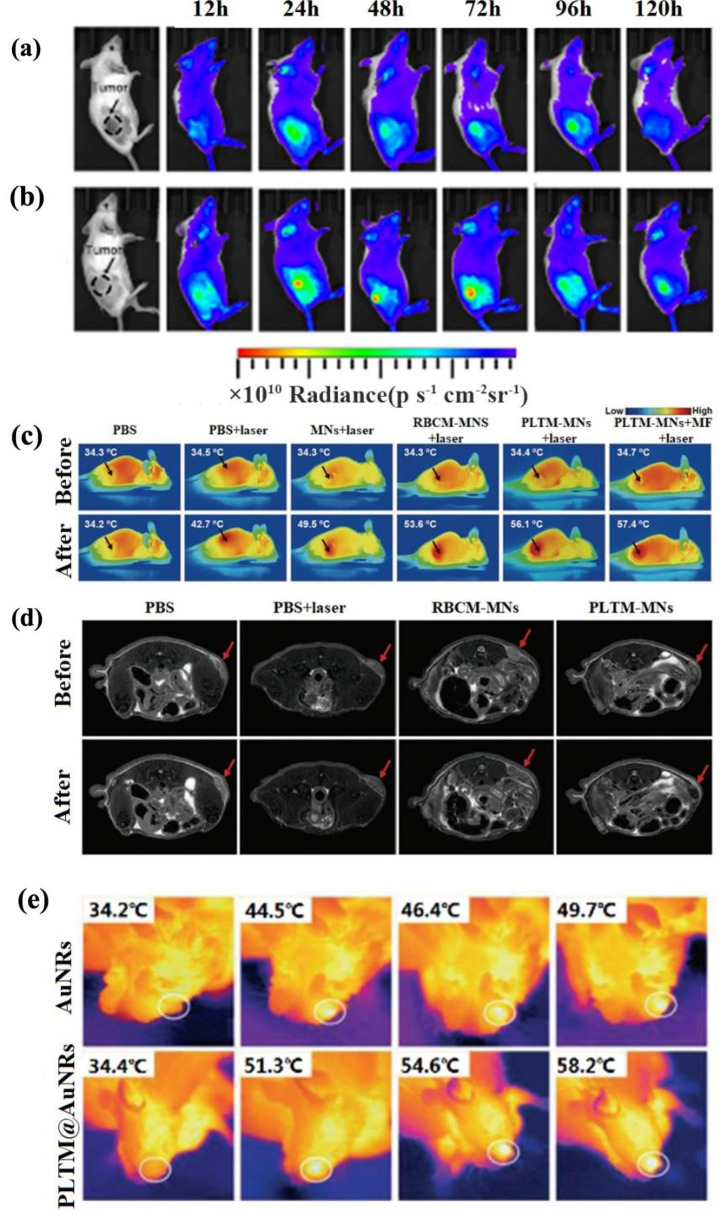
IR780 embedded within the nanoparticles is used as the photothermal agent, which could also be applied in vivo fluorescence imaging of 4T1 tumor-bearing mice at 12, 24, 48, 72, 96, and 120 h after intravenous injection of **(a)** bare core INPs and **(b)** PLTM-INPs lied in NIR fluorescence imaging. **c** Comparison of representative in vivo IR of mice bearing MCF-7 tumor injected with different components after laser irradiation for 5 min. **d** magnetic resonance imaging contrast after all kinds of nanoparticles injected with the same amount. Red arrows indicated the sites of MCF-7 tumors in mice. **e** After PTT treatment with different kinds of nanoparticles, the highest tumor temperature in treated mice increased. White circles indicate the tumor sites. Reprinted with permission from Refs [32, 103]

Compared with active targeting, passive targeting is more widely used. Pei et al*.* improved the precise delivery of drugs with the participation of PLTM. They prepared IR780 (a typical NIR fluorescent dye) PLGA and DOX for IR780@PLGA/DOX NPs by single emulsification to treat breast cancer [102]. NPs that contained drugs and photothermal agents wrapped with natural PLTM could achieve no recognition and little clearance by the immune system. As shown in Fig. 12a, b, the photothermal fluorescence imaging in vivo indicated that the PLTM-INPs could circulate in the blood for a more extended period and accumulate more at the tumor sites compared with the bare core INPs, which released more antitumor drugs to achieve better PTT results. Fluorescence imaging of tumors in vivo could be obviously observed from 12 to 120 h after injection. It is worth mentioning that the tumors on mice models treated with PLTM-INPs thoroughly vanished without recurrence during 18 d observation period [102]. This study provided a new idea to design photothermal agents and drug delivery systems by loading them into PLTM vesicles.

To take advantage of the long circulation and cancer-targeting ability of PLTM, Liu et al*.* developed PLTM-camouflaged Fe_3_O_4_ magnetic NPs (PLTM-MNs) that inherited tumor targeting molecules from PLTM and the photothermal conversion and magnetic reactions of the Fe_3_O_4_ NPs [103]. In a mouse model, PLTM-MNs had similar blood retention to RBCM-MNs within 48 h. More importantly, PLTM-MNs uptake was lower in the liver and spleen rich in RES and macrophages compared with MNs and RBCM-MNs, while the amount of internalization was higher in tumor sites. Quantitative measurements of mice tissues by inductively coupled plasma atomic emission spectrometers showed PLTM-MNs had a lower uptake and better tumor-targeting ability. The results showed that PLTM could be used to enhance PTT in cancer therapy. The tumor-bearing mice injected with PLTM-MNs and exposing laser irradiation regardless of external magnetic field (MF) exhibited the highest tumor temperature increase from 34.4 to 56.1 ℃ (Fig. 12c). Moreover, the T_2_-weighted MR images confirmed that PLTM-MNs possessed better tumor accumulation capability than other groups, which indicated that it was able to run MRI examination to achieve personalized diagnosis and treatment of tumors (Fig. 12d) [32].

Liu et al*.* did experiments with gold nanoclusters wrapped with PLTM, which took advantage of specific adhesion to injured vessels and tumor tissues of PLTM [77]. The results showed that the PLTM@AuNRs system circulatory performance of the 48 h was more stable and lasting than the exposed AuNRs in vivo, while the major organ microphysiological systems accumulated less. Notably, compared with the absence of laser irradiation, the tumor site accumulated more AuNRs after laser irradiation, which may be explained as that the PLTM@AuNRs had self-healing ability of the tumor injury site by active targeting. Therefore, after each treatment, the maximum tumor temperature of PLTM@AuNRs treated mice increased continuously, and positive feedback appeared through this self-reinforcing characteristic of PLTM@AuNRs, resulting in an enhanced PTT effect, which was proved by the representative in vivo IR thermal images before and after each treatment (Fig. 12e). This work provided a new angle on the design of biomimetic PLTM-INPs for personalized diagnosis and therapy of various diseases with injured vessels. However, controlling temperature increase to avoid burning normal tissues is a tricky problem since INPs that have a photothermal property can also accumulate apart from tissues with injured vessels.

**Fig. 13:**
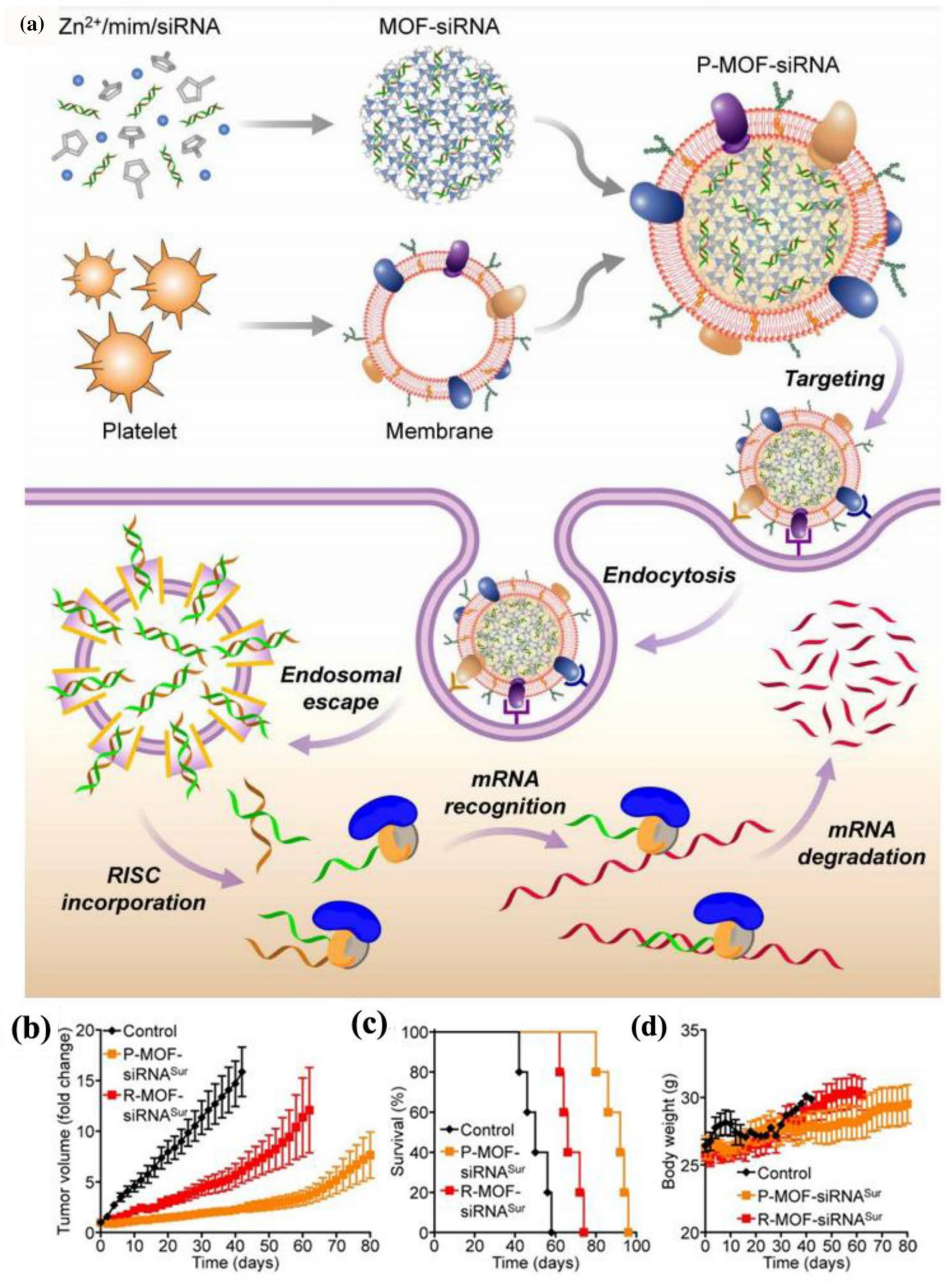
**a** Platelet membrane–coated siRNA-loaded MOFs (P-MOF-siRNA) for gene silencing. **b** Growth kinetics of SK-BR-3 tumors implanted subcutaneously into nu/nu mice and treated intravenously with P-MOF-siRNA or R-MOF-siRNA every 3 days for a total of four administrations (*n* = 5; mean ± SEM). **c** Survival of the mice over time (*n* = 5). **d** Body weight of the mice in **(a)** over time (*n* = 5; mean ± SD). Reprinted with permission from Refs [104]

Apart from encapsulating small-molecule anti-cancer INPs, such as DOX and Fe_3_O_4_ NPs, PLTM-INPs were also utilized for delivering gene to achieve gene therapy for cancer, such as using small interfering RNA (siRNA) to silence tumor-relevant genes. In one design, Zhuang et al*.* loaded the synthesized siRNA into zeolite imidazole ester skeleton-8 (zif-8) of the porous metal–organic skeleton (MOF) INPs and then coated PLTM onto this inorganic nanoplatform to form P-MOF-siRNA (Fig. 13a) [104]. The P-MOF-siRNA showed the lowest immunogenicity, and a binding test showed the highest affinity to tumor cells than the RBC membrane-camouflaged NPs (R-MOF-siRNA). The nude mice treated with P-MOF-siRNA exhibited a more robust tumor growth inhibition rate and a higher survival rate than those treated with bare INPs (Fig. 13b–d). Overall, Zhuang et al*.* successfully construct a biomimetic nanoplatform for effective siRNA delivery. In consideration of long-term toxicity use in human patients, the nanoplatform utilizing cell membrane-camouflaged MOF as delivery vehicles could help expand the field of nucleic acid-based therapies, such as immune modulation and gene therapy, since any kind of RNA molecule can be easily loaded into the porous MOF. It is also worth mentioning that multiple anti-cancer nanomedicines could be encapsulated into the same MOF to achieve multifunctions.

PLTM-INPs can not only increase tumor sites accumulation with the ability of CTC-targeting, but also reduce systemic toxicity. With rich sources, low cost, simple extraction, and low immunogenicity, PLTM-INPs have the potential to be one of the best candidates for the biomimetic system for cancer therapy.

## Hybrid cell membrane-camouflaged inorganic nanoparticles (HCM-INPs)

After the previous discussion, cell membrane-camouflaged INPs possess various unique characteristics, but in some circumstances, the single-cell membrane-camouflaged INPs are not enough to meet ideal demands. For instance, the RBCM cannot target the tumor for lack of associated tumor adhesive molecules [100]. Compared with the single-cell membrane-camouflaged INPs, the HCM-INPs concentrate multiple functions from different source cells on one platform. It is a relatively new method that gives INPs specialized functions. It can inherit the targeting ability of CCM, as well as the immune evasion ability of RBC. New functions can be developed through the combination of different types of cell membranes. And on account of different tumors, HMC-INPs can enable the implementation of personalized therapies. Since the first synthesis of HCM-INPs by Zhang’s group, there have been many HCM-INPs platforms getting designed for different aspects [104–106].

**Fig. 14:**
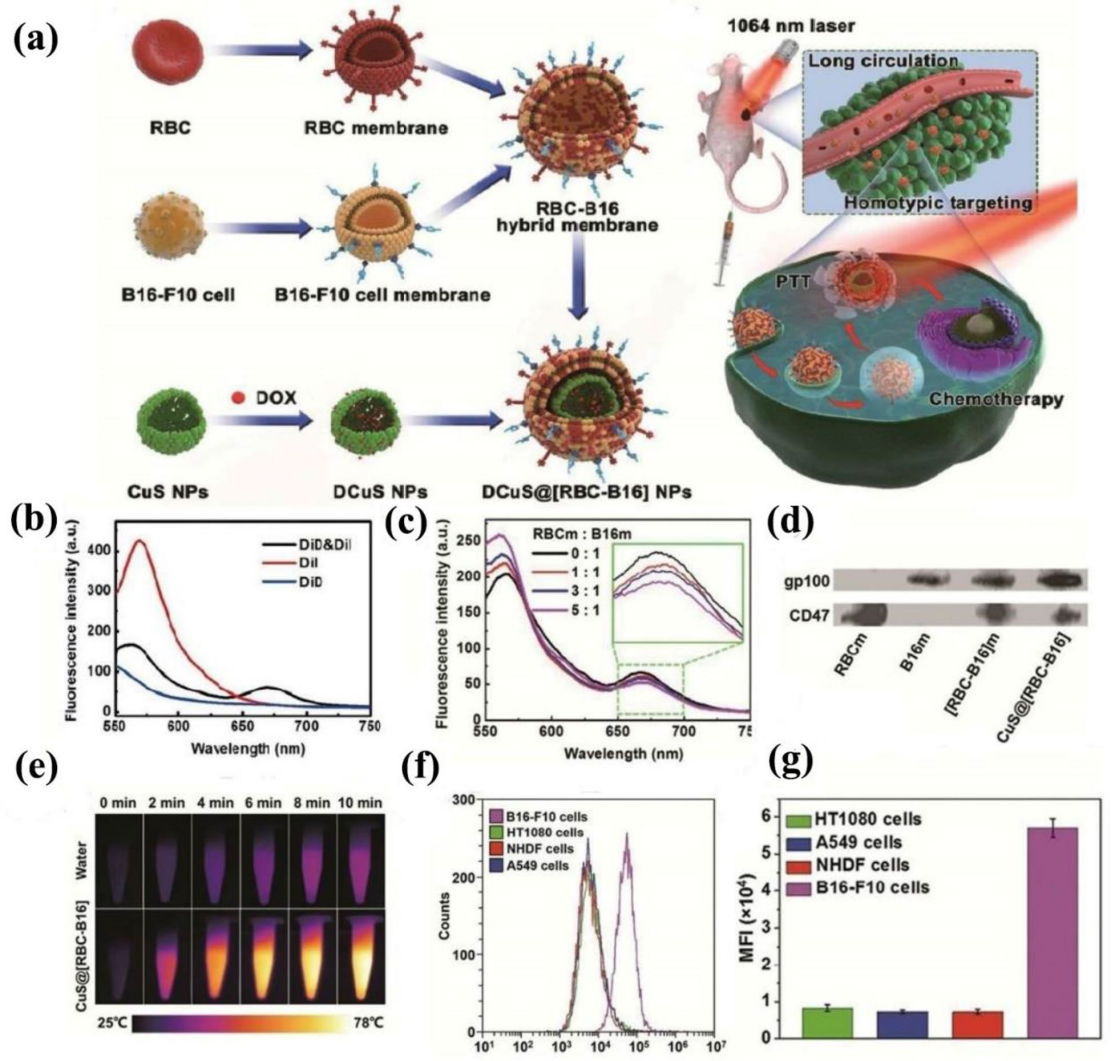
**a** The process of membrane fusion and the hybrid membrane was coated on the CuS NPs and the brief principle of tumor treatment. **b** FRET pair dyes of DiD and DiI, or single DiD or DiI, were employed to label the B16-F10 cell membrane. **c** The FRET with the addition of RBCM. **d** Western blot protein analysis of CD47 and gp120 in different systems. **e** Infrared thermal imaging of water and CuS@[RBC-B16]. Flow cytometric profiles (**f** and **g)** Mean fluorescence intensity of the four cell lines B16-F10, HT1080, NHDF, A549 upon 4 h incubation with DiI dyed CuS@[RBC-B16] NPs. Reprinted with permission from Refs [70]

Wang et al*.* proposed CuS NPs that were coated by fusing RBCM with B16-F10 CCM (CuS@[RBC-B16]NPs) [70]. CuS exhibits excellent drug loading efficiency and the ability of photothermal conversion, which has the potential to become a multifunctional platform. It showed highly specific self-recognition and visible longer circulatory time after being camouflaged by the RBC-B16 membrane (Fig. 14a). To characterize the HCM, a Förster resonance energy transfer (FRET) pair dyes, DiD and DiI were used for indicating the fusion of the two kinds of cell membranes. With the addition of the RBCM, the fluorescence intensity changed accordingly. The two dyes become scattered with the fusion of the RBCM (Fig. 14b, c). As shown in Fig. 14d, the gp100 (Characteristic molecules of B16-F10 membrane) and CD47 (Characteristic molecules of RBCM) could be found in the [RBC-B16] membrane and CuS@[RBC-B16] NPs groups making use of the Western Blot analysis. CuS@[RBC-B16] NPs showed superior efficiency and persistence in photothermal conversion (Fig. 14e). As shown in Fig. 14f, g, after being incubated with DiI-dyed CuS@[RBC-B16] NPs, the B16 showed higher fluorescence intensity than other cells in flow cytometric profiles. This study gave a new insight to design personalized anticancer nanoplatform by combining RBCMs with homotypic CCMs to coat the surface of the INPs. This work gives an inspiring idea and method of hybridizing different types pf cell membranes to achieve combination of multiple biological functions, and a series of strategies were shown to characterize HCM-INPs, providing guidance for the following research on HCM-INPs. Since RBCM is easy and cheap to require, more attempts can carry out to hyrid RBCM with various natural cell membranes to endow CMCINPs with a variety of biological functions.

**Fig. 15:**
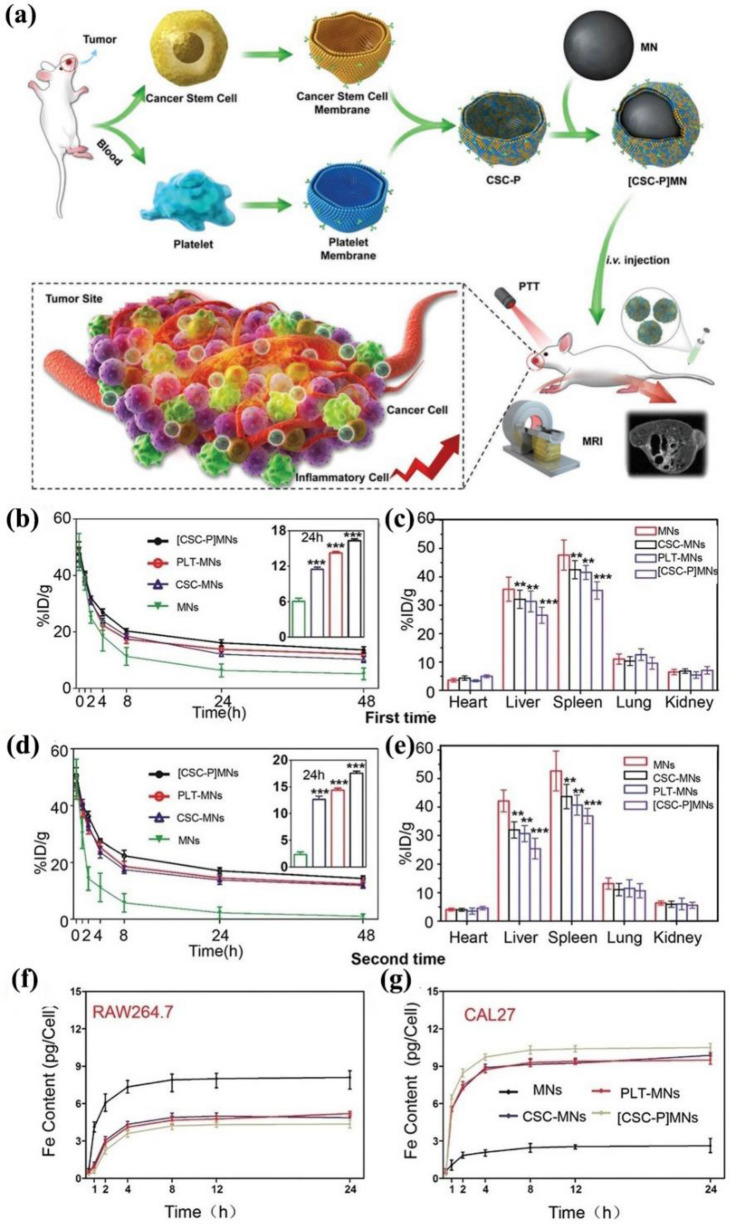
**a** The synthesis process of [CSC-P]MNs and the principle of treatment. **b** The clearance after the first injection. **c** The accumulation amount of different NPs in different organs after the first injection. **d** The clearance rate of different NPs after the second injection. **e** The accumulation in different organs after the second injection. **f** The Fe content in RAW264.7 macrophage-like cells in cellular uptake experiment. **g** The Fe content in CAL27 cancer cells in cellular uptake experiment. Reprinted with permission from Refs [108]

The Fe_3_O_4_ NPs are frequently used and clinically acceptable to conduct the PTT [107]. However, the odd is that the Fe_3_O_4_ NPs accumulate little in the tumor due to the immune clearance and lack of the ability to target. Moreover, the first injection of INPs will cause a quick clearance of the following injection, and this circumstance is called the accelerated blood clearance phenomenon (ABC phenomenon). To deal with these defects, Bu et al*.* developed hybrid cancer stem cell-platelet membrane-camouflaged Fe_3_O_4_ NPs ([CSC-P]MNs) as shown in Fig. 15a to solve the ABC phenomenon during the treatment of the head and neck squamous cell carcinoma [108]. The detection of the Fe can reflect the circulation time of Fe_3_O_4_ NPs. As shown in Fig. 15b, d, the circulation time of [CSC-P]MNs was longer after the second injection compared with other groups, and in Fig. 15c, e, the accumulation of [CSC-MNs] in the liver and spleen changed little, which indicated that the ABC phenomenon was mitigated. As shown in Fig. 15f, g, through the cellular uptake experiments, the CAL27 cancer cells emerged with a higher uptake rate for [CSC-P]MNs compared with RAW246.7 macrophage-like cells, reflecting that [CSC-P]MNs had a good tumor-targeting ability. In conclusion, the [CSC-P]MNs were reliable HCM-camouflaged nanoplatforms for immune evasion, magnetic resonance imaging, tumor targeting, and PTT. The combination of platelet and cancer stem cell membrane can overcome multiple defects of INPs at the same time, such as the ABC phenomenon, ensuring INPs play a durable and efficient function. However, determining the optimal mass ratio of different cell membranes to realize the most efficient functions of HCM-INPs is a time-consuming process. Meanwhile, the reconstruction of HCM might change the distribution of the surface cell membrane proteins, which might influence its functions.

## Conclusion and perspective

This review highlights the recent advances in CMCINPs for cancer treatment and several strategies to prepare the CMCINPs, which are summarized in Table 2. Compared with the bare inorganic cores, the CMCINPs overcame many challenges, including fast immune clearance, limited targeting ability, toxicity to the human body, and essentially retaining the features of INPs, which could realize the personalized and efficient tumor treatment with fewer side effects. After coating with the different cell membranes, the INPs can also inherit various functions of the cell membranes. The RBCM-INPs can prolong blood circulation time, reduce RES uptake ratio, and escape immune clearance. The MSCM-INPs can target the tumor and damaged tissue. The CCM-INPs have been widely used for homogeneous tumor targeting. The WBCM-INPs have been proven to have the ability of inflammation sites targeting, specific tumor-targeting, detection of cancer cells, and transendothelial migration. The PLTM-INPs have been reported to apply in drug delivery, detoxification, and CTC-targeting. The HCM-INPs can fuse different cell membranes’ functions to construct a versatile therapeutic platform.

**Table 2:** Summary of CMCINPs in this review

Type	Key features	Origin of shell material	Core material	Preparation strategy	Cargo loaded	Applications	Ref
RBCM-INPs	Long circulation; Immune escape	RBC	Fe_3_O_4_	Extrusion	/	MRI; Anti-tumor drug delivery; Hyperthermia; Tissue repair	[47]
			MNC	Sonication	Cyp	Fluorescence imaging; MRI; PTT	[38]
			UCNPs	Extrusion	FA	Fluorescence imaging; PDT	[49]
			MSNs	Extrusion	Dox/Ce6	Fluorescence imaging; Drug delivery	[59]
			AuNCs	Extrusion	/	PTT	[109]
			MNs	Microfluidic Electroporation	/	PTT; MRI	[48]
CCM-INPs	Homologous targeting; Immune escape	UM-SCC-7; H22; HeLa; COS7	MNP	Extrusion	DOX	Drug delivery; MRI	[63]
		KB	Gold	Extrusion	/	PTT; Radiotherapy	[64]
		B16-F10	H-MnO2	Stir/Co-incubation	Ce6;GOx	PDT; Starvation therapy	[77]
MSCM-INPs	Tumor-homing affinity; Easy expansion in vitro	MSC	UCNPs	Extrusion	ZnPc;MC540	PDT	[81]
			SPIO	Sonication	STM	Drug-targeted delivery; Magnetic hyperthermia therapy; MRI	[82]
WBCM-INPs	Inflammation targeting; Reducing opsonization	Macrophage	AuNS	Extrusion	/	PTT; Bio-imaging	[86]
		Leukocyte cells (THP-1)	GNS	Sonication	DOX	PTT; Drug delivery	[91]
		Leukocyte cells	MNC	Sonication	/	CTC detection	[96]
PLTM-INPs	Low immunogenicity; Cancer targeting ability	Platelet	MNs	Extrusion	/	MRI; PTT	[32]
			AuNRs	Microfluidic Electroporation	/	PTT	[103]
			MOF	Extrusion	siRNA	Tumor-associated gene silencing	[104]
HCM-INPs	Immune escape; Homogenous targeting	RBC/B16-F10	CuS	Sonication	DOX	Drug delivery; PTT	[70]
		Platelet/Cancer cell/stem cell	MNs	Extrusion/Sonication	/	PTT; MRI	[108]

To the best of our knowledge, there are a series of clinical trials based on cell-derived vesicles are underway, such as a Phase I clinical trial from 2011 investigating the ability of plant exosomes to deliver curcumin to colon tumors and normal colon tissue (NCT01294072), and a Phase II clinical trials from 2013 recruiting 30 malignant ascites or pleural effusion patients to evaluate the safety and effectiveness of tumor cell-derived MVs (NCT01854866) [110]. However, none of the clinical trials are about CMCINPs applicated in cancer therapy. Moreover, there are little survey based-on cell membrane-derived vesicles conducted to promote clinical translations for medical applications. Up to now, drug candidates concentrated on cell membrane vesicles barely derived from red blood cells and macrophages, which is applicated for MRSA pneumonia (No. CTI-005), sepsis (No. CTI-111), coronavirus (No. CTI-118), cytokine release syndrome (No. CTI-156), and inflammatory bowel disease (No. CTI-168), which utilizes the surface porous structure acting as a nanosponge to absorb the toxins, bacteria, cytokines, virus, and so on. All these clinical trials have not been come into Phase I study. Though the cell membrane-camouflaged technique has got some developments, there are still some problems unsolved that obstruct the clinical translation of the CMCINPs, and issues primarily focus on cell source, biosafety, mechanisms of action at biointerfaces, the preparation of the CMCINPs, and the optimization of the production process.

The source of the cells is one of the most difficulties which confine the works in the laboratory. For some cell types, it is hard to acquire sufficient cell membrane vesicles from the patient and thus restrict the yield’s development. Moreover, when using the cells from donors, the blood type and the immune rejection are necessary to be considered. There has been scant report about the in vivo reaction between CMCINPs and the human system, such as hemolytic reactions, using the RBCM-INPs with different types of ABO antigen or other CMCINPs with various human leukocyte antigens (HLA). The use of the cancer cell line is also a considerable choice, but the potential carcinogenicity of the nucleic acids might occur since they are not removed thoroughly.

Additionally, the challenges also include biosafety, for which it is hard to make sure all of the cell membrane vesicles are utilized to coat onto INPs, and the uncoated INPs will induce an intense immune reaction so that the high output membrane coating technology is urgently needed. More clinical research, like pharmacokinetics and post-marketing surveillance, need to be conducted with bare inorganic cores.

Most of the current understanding of the mechanism of action of CMCINPs tends to concentrate on the interactions of the surface molecules of the cell membrane, especially the cluster of differentiation or the shadowing of INPs surfaces. Nevertheless, the detailed characterization of the molecules confines the research of the concrete mechanism in plenty of research. Furthermore, whether the interaction between INPs and cell membranes can change the conformation of the molecules or not has not been demonstrated in most research. Moreover, although there are many coating methods, the mechanism of some methods and the concrete parameter control are not clear, which restricts the standardized production. With the development of cell biology, different molecular mechanism and other surface molecules such as carbohydrates should be discussed.

The standardized production process is necessary to achieve large-scale production and clinical translation. The fabrication process of CMCINPs is relatively complex and expensive, and every step should be performed in an aseptic environment to ensure the quality of the products. Meanwhile, the short storage time of CMCINPs makes it challenging to meet the needs of clinical applications. The cell source mentioned above is also one of the challenges. All these factors limit the clinical translation of CMCINPs.

As an emerging technique, the great potential of CMCNIPs in medical care will undoubtedly promote researchers to explore more in the future, and it will be a boon to human health. Even though there are still many difficulties ahead, from the current work progress, we can promise that the CMCINPs have great potential in therapy and diagnosis for cancers, potentially bringing a significant change in current cancer treatment modalities.

## Data Availability

Not applicable.
